# Adenosine Deaminases Acting on RNA (ADARs) and Viral Infections

**DOI:** 10.1146/annurev-virology-091919-065320

**Published:** 2021-04-21

**Authors:** Christian K. Pfaller, Cyril X. George, Charles E. Samuel

**Affiliations:** 1 Division of Veterinary Medicine, Paul-Ehrlich-Institute, Langen 63225, Germany; 2 Department of Molecular, Cellular, and Developmental Biology, University of California, Santa Barbara, California 93106, USA

**Keywords:** adenosine deaminase acting on RNA, ADAR, autoimmunity, double-stranded RNA, dsRNA, innate immunity, interferon, RNA editing

## Abstract

C6 deamination of adenosine (A) to inosine (I) in double-stranded RNA (dsRNA) is catalyzed by a family of enzymes known as ADARs (adenosine deaminases acting on RNA) encoded by three genes in mammals. Alternative promoters and splicing produce two ADAR1 proteins, an interferon-inducible cytoplasmic p150 and a constitutively expressed p110 that like ADAR2 is a nuclear enzyme. ADAR3 lacks deaminase activity. A-to-I editing occurs with both viral and cellular RNAs. Deamination activity is dependent on dsRNA substrate structure and regulatory RNA-binding proteins and ranges from highly site selective with hepatitis D RNA and glutamate receptor precursor messenger RNA (pre-mRNA) to hyperediting of measles virus and polyomavirus transcripts and cellular inverted *Alu* elements. Because I base-pairs as guanosine instead of A, editing can alter mRNA decoding, pre-mRNA splicing, and microRNA silencing. Editing also alters dsRNA structure, thereby suppressing innate immune responses including interferon production and action.

## INTRODUCTION: ADARs AND THEIR REGULATION

**Figure 1:**
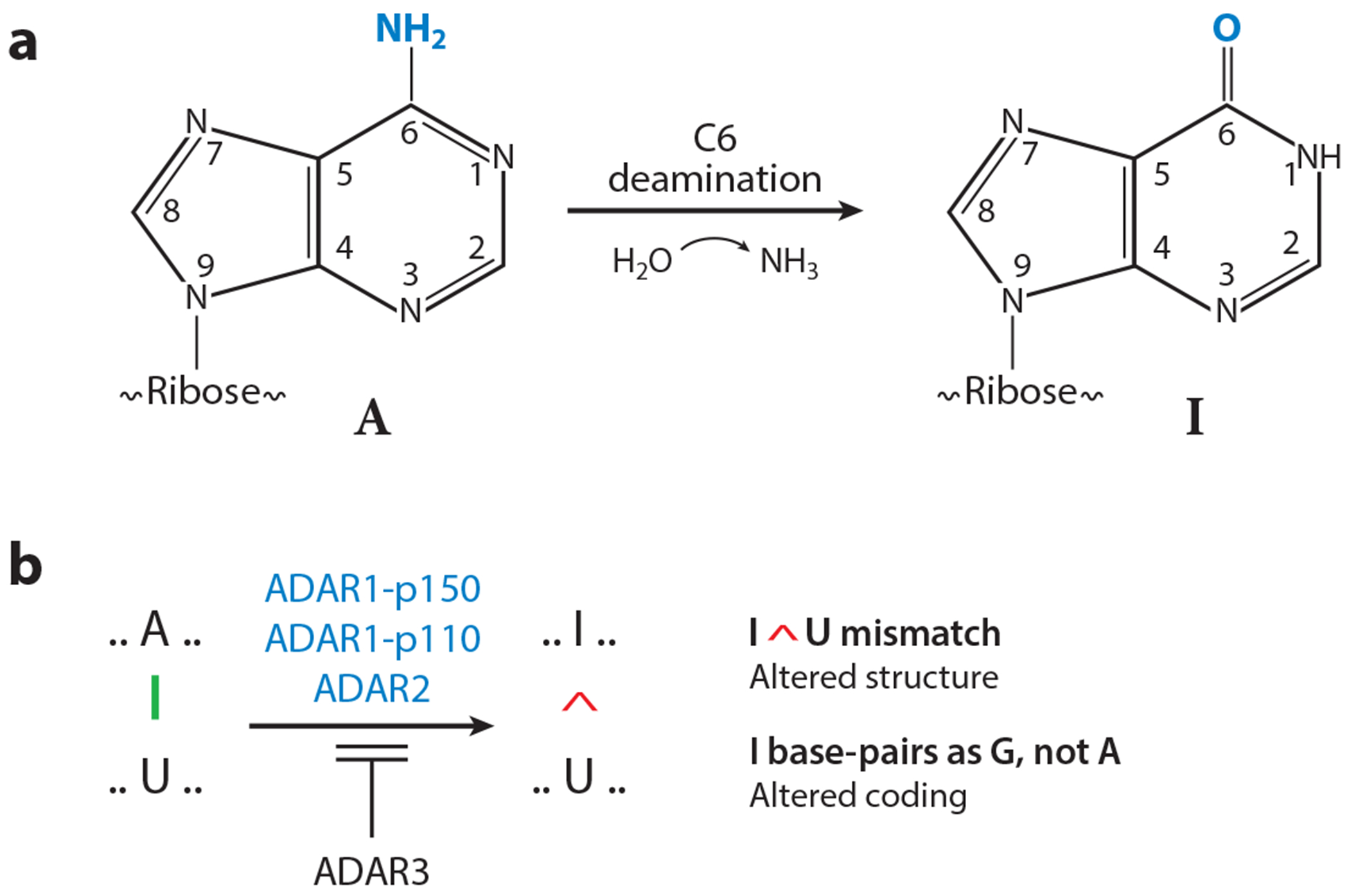
Deamination of A in dsRNA by ADARs. (*a*) C6 deamination of A yields I. (*b*) Adenosine deamination in dsRNA is catalyzed by ADARs. In mammals, there are four ADARs, one interferon-inducible (ADAR1-p150) and three constitutively expressed (ADAR1-p110, ADAR2, and ADAR3). ADAR1-p150, ADAR1-p110, and ADAR2 possess enzymic activity, whereas ADAR3 lacks deaminase activity but can inhibit active ADARs. Abbreviations: A, adenosine; ADARs, adenosine deaminases acting on RNA; dsRNA, double-stranded RNA; G, guanosine; I, inosine. Figure adapted from Reference 15.

Adenosine deaminases acting on RNA, known as ADARs (1), were discovered during antisense RNA studies in *Xenopus* (2, 3). Sense-antisense double-stranded (ds) RNA structures were destabilized based on their altered mobility during native gel electrophoresis and increased sensitivity to nuclease degradation by single-strand selective RNase. The activity responsible for these changes in dsRNA structure catalyzed the C6 deamination of adenosine (A), a covalent modification that produces inosine (I) (Figure 1a). This process now is called A-to-I editing (4–8). Because I generated by ADAR base-pairs as guanosine (G) instead of A, the deamination effectively alters the RNA sequence by making a purine nucleotide substitution of I (= G) for A (Figure 1b). Such substitutions can alter coding, as I base-pairs with cytosine (C) instead of uracil (U). Substitution of I for A also can alter RNA structure because an I-U mismatch pair is less stable than an A:U pair (9).

ADARs are different from adenosine deaminases (ADAs) that also catalyze the C6 deamination of A. The substrate for ADA is the free nucleoside A, whereas ADARs deaminate A present in dsRNA. ADA is an important enzyme of the purine salvage pathway. Loss of ADA activity results in severe combined immunodeficiency (10). Dysregulation of ADAR1 activity also results in human diseases including Aicardi-Goutières syndrome, an autoimmune disorder and interferonopathy (11); bilateral striatal necrosis dystonia (12); and dyschromatosis symmetrica hereditaria, a skin pigmentation disorder (13).

This review concerns the regulation and functions of ADARs and the roles they play during viral infections. dsRNA has a long history in virology and innate immunity, the cornerstone of which is the interferon (IFN) response (14–16). A-to-I editing of dsRNAs by ADARs triggers substantial functional changes, in both uninfected and virus-infected cultured cells and intact animals. These changes are caused by editing-induced alterations in RNA sequence and structure. A-to-I editing effectively substitutes a G for an A and hence causes a change in RNA sequence. Such substitutions may affect sequence-dependent processes such as messenger RNA (mRNA) decoding, precursor mRNA (pre-mRNA) splicing, microRNA silencing, and RNA-dependent replication by viral polymerases. And because an I-U mismatch pair is less stable than an A:U base pair, A-to-I editing also may alter RNA structure-dependent sequence-independent processes such as dsRNA sensing.

There are three *ADAR* genes in mammals (1): *ADAR1* (*ADAR*), *ADAR2* (*ADARB1*), and *ADAR3* (*ADARB2*). These genes encode three enzymically active ADAR proteins: ADAR1-p150 that is IFN inducible, and ADAR1-p110 and ADAR2 that are constitutively expressed. A fourth ADAR protein, ADAR3, does not possess demonstrable deaminase activity. ADAR1 and ADAR2 deaminate both viral and cellular dsRNAs. The biologic effects of the resultant A-to-I substitutions range from antiviral to proviral to none, with the outcome dependent upon the specific virus, the particular ADAR deaminase, and the physiologic conditions (15, 17–19). We consider here the three ADARs, what they are, and what they do, and we describe examples of their effects on virus-host interactions.

## ADAR1

**Figure 2:**
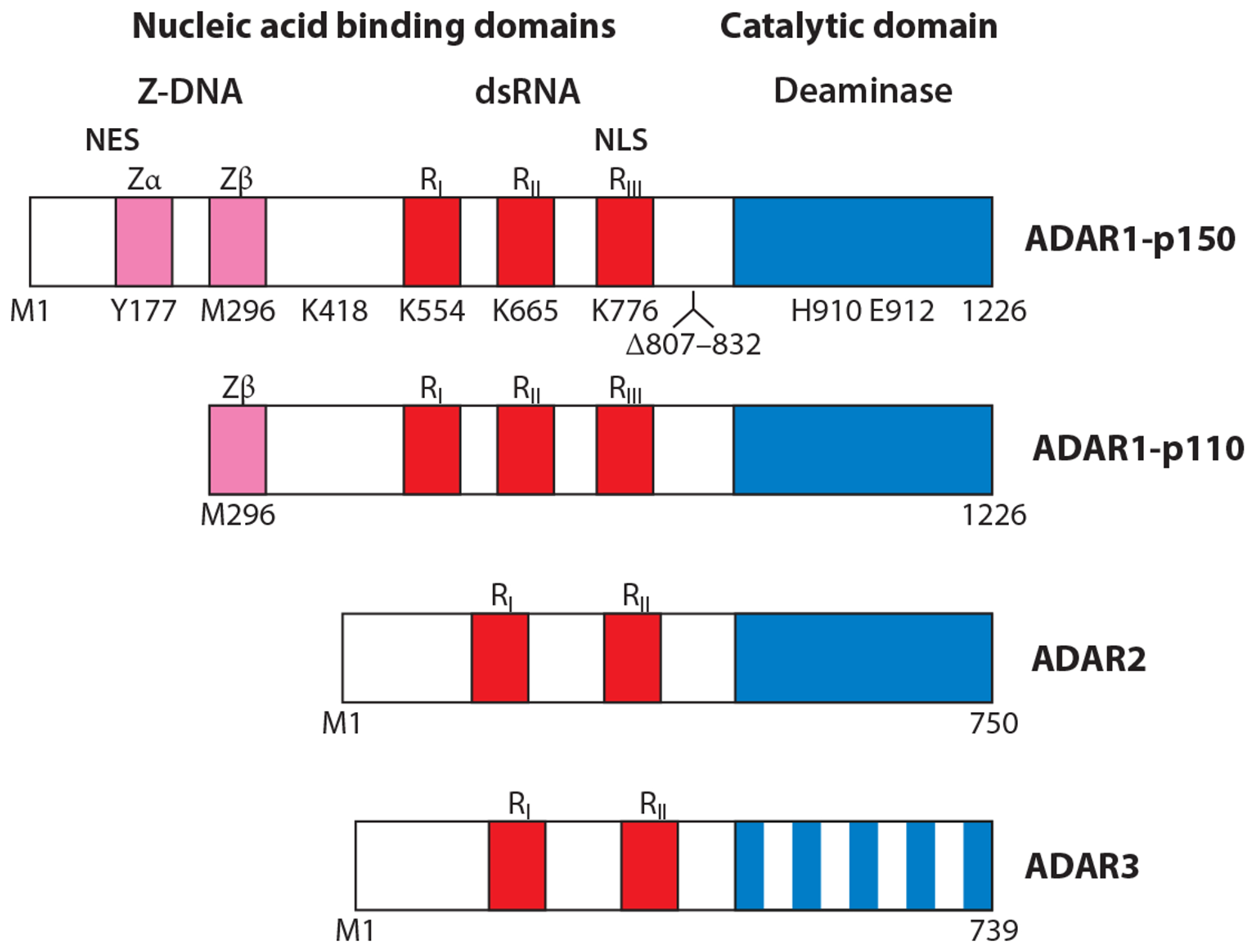
Domain organization of ADAR proteins. The deaminase catalytic domain (*blue*) is present in the C-terminal region of the active ADAR1 and ADAR2 enzymes and the inactive ADAR3 (*blue columns*) protein. Repeated dsRNA-binding domains (*red*) are designated R_I_, R_II_, and R_III_. The N-terminal region of ADAR1-p150 possesses repeated copies of the Z-DNA-binding domains (*pink*), designated Zα and Zβ. Inducible ADAR1-p150 initiates at M1 and lacks amino acid residues 807–832 due to alternative transcript splicing; constitutive ADAR1-p110 initiates at M296 and includes amino acid residues 807–832. Human ADAR1 is sumoylated at K418; mutation of K554, K665, and K776 impairs dsRNA binding activity of the respective R domain; and mutation of Y177 of Zα impairs DNA binding activity. H910 and E912 are essential residues of the CHAE deaminase catalytic core. Abbreviations: ADARs, adenosine deaminases acting on RNA; dsRNA, double-stranded RNA; NES, nuclear export signal; NLS, nuclear localization signal. Figure adapted from Reference 7.

**Figure 3:**
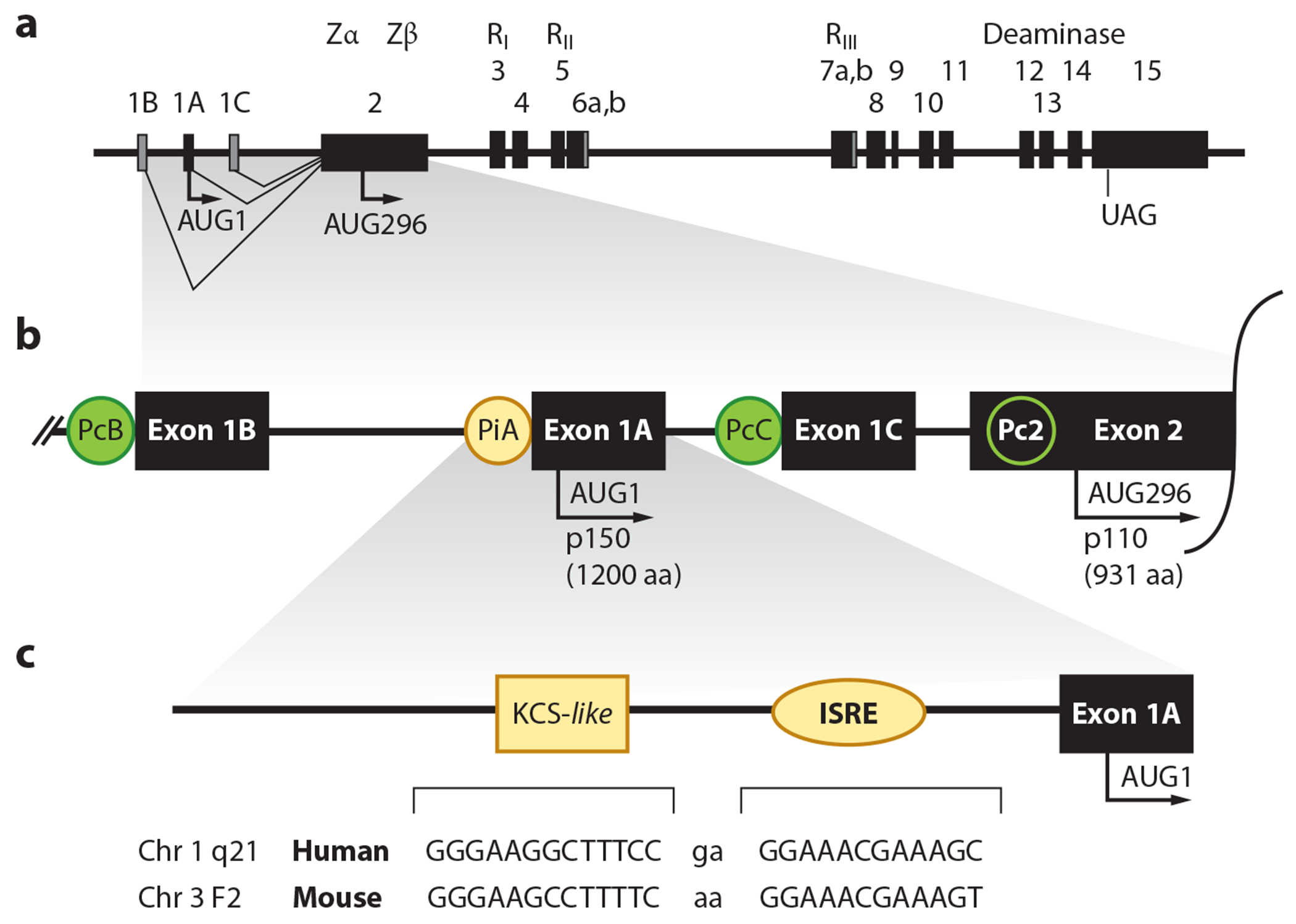
Organization of the human *ADAR1* gene. (*a*) Exon-intron organization of the ~40-kbp human *ADAR1* gene found on chromosome 1q21. Exons 1–15 are indicated by black boxes and include alternative exons 1A, 1B, and 1C and alternative exons 6a and 6b and 7a and 7b. Introns and 5’- and 3’-flanking regions are indicated by solid lines. (*b*) Alternative promoters are indicated by circles and include PiA (*yellow*), which is IFN inducible, and PcB, PcC, and Pc2 (*green*), which are constitutively active. The PiA promoter drives expression of alternative exon 1A-, exon 7b-containing transcripts that encode the 1,200-aa ADAR1-p150 protein that initiates from AUG1 present in exon 1A. Alternative exons 1B and 1C do not contain an AUG; constitutively active promoters drive expression of transcripts that initiate translation of the 931-aa ADAR1-p110 protein from the in-frame AUG296 codon present in exon 2. (*c*) The human *ADAR1* and mouse *Adar1* genes possess highly conserved PiA promoter elements (*yellow*) that drive the IFN-inducible expression of exon 1A-containing transcripts: a 12-bp ISRE found in type I IFN-inducible genes and an adjacent 13-bp KCS-*like* element, which is also found in the protein kinase R promoter. Abbreviations: ADARs, adenosine deaminases acting on RNA; IFN, interferon; ISRE, interferon-stimulated response element; KCS, kinase-conserved sequence. Figure adapted from Reference 17.

There are two isoforms of ADAR1 (Figure 2): p150 is IFN inducible and found in both the cytoplasm and nucleus; p110 is constitutively expressed and predominantly if not exclusively found in the nucleus (20). The human *ADAR1* gene maps to chromosome 1q21 (21), and mature transcripts include 15 exons (Figure 3). The ADAR1 consensus complementary DNA (cDNA) sequence from human cells possesses an open reading frame (ORF) of 1,226 aa (20, 22, 23).

ADAR1 is ubiquitously expressed and inducible by microbial infections (5, 6, 15, 24–27). Expression is driven by alternative promoters (Figure 3), one IFN inducible (PiA) and the others constitutively active (PcB, PcC, and Pc2) (28–30). Alternative splicing of the IFN-inducible transcripts produces mRNA beginning with alternative 5′-exon 1A that possesses AUG1, the Met initiation codon for p150 synthesis. Exon 1A-containing ADAR1 transcripts typically have alternative exon 7b and encode a 1,200-aa p150 protein. Constitutively expressed mRNA begins with alternative 5’-exon 1B (or 1C), which lacks an AUG and typically has exon 7a. These transcripts encode a 931-aa p110, whose translation is initiated at AUG296 in exon 2 (28–30). Exon 1A-containing mRNA is optimized to express both p150 and p110 by a leaky scanning translation initiation mechanism (26). However, the majority of p110 is synthesized from exon 1B- or 1C-containing mRNAs. The promoter and exon organization of the mouse *Adar1* gene (24, 27, 31, 32) is conceptually similar to that of the human *ADAR1* gene; mouse p150 is 1,152 aa, and mouse p110 is 903 aa.

Induction of ADAR1-p150 by IFN occurs through Janus kinase-signal transducers and activators of transcription signaling (33) by the transcriptional activator complex STAT1-STAT2-IRF9. The inducible PiA promoter for the human (28) and mouse (34) genes possesses a consensus interferon-stimulated response element (Figure 3). The STAT2-dependent, IRF9-dependent induction of *Adar1* by IFN differs between mouse and human cells in the stringency of the requirement for STAT1 (34, 35). The PiA promoter also possesses a kinase-conserved sequence–like element (Figure 3) initially identified in the protein kinase R (PKR) promoter that enhances transcriptional activity through Sp factor binding (17).

The p150 and p110 ADAR1 proteins each have three copies of the dsRNA-binding domain (R) in their central region and the deaminase catalytic domain in the carboxy (C)-terminal region (7, 8, 20, 22, 23, 36) (Figure 2). R domains bind right-handed, A-form dsRNA in a sequence-independent manner. ADAR1-p150 is an amino (N)-terminally extended version of ADAR1-p110; the additional 295-aa sequence of human ADAR1-p150 includes the Z-DNA/Z-RNA binding (Z)-domain Zα that binds left-handed, Z-form dsDNA and dsRNA. Recognition of CpG repeats and also non-CpG repeats in a broad range of RNA sequences by Zα is accompanied by destabilization of neighboring A-form regions (37). ADAR1 domains for dsRNA-binding and deaminase catalytic activity are functionally distinct (38). Mutation of lysines K554, K665, and K776 present in the ADAR1 dsRNA-binding domains impairs binding of dsRNA (Figure 2). Mutation of histidine H910 and glutamic acid E912 in the catalytic domain CHAE sequence of ADAR1 destroys deaminase activity. Sumoylation at K418 reduces ADAR1 deaminase activity (39). Protein kinase B–dependent phosphorylation of ADAR1-p110 at threonine T738, and ADAR2 at T553, reduces deaminase activity; the phosphomimic mutants T738D and T553D show reduced editing activity (40). Stress-activated phosphorylation of ADAR1-p110 by mitogen-activated protein kinases promotes Staufen1-mediated mRNA decay (41). Finally, Y177 is an essential tyrosine residue for function of the Zα domain of p150 (36, 42).

A-to-I editing sites have been identified by multiple strategies, including RNA sequencing. The vast majority of sites occur in repetitive noncoding sequences exemplified by *Alu* sequences in the human transcriptome; ADAR1 is largely responsible for most of these editing events, including the hyperediting of viral and cellular RNAs (6, 8, 25, 43). Editing analysis of total RNA from ADAR1 null cells reconstituted with either p150 or p110 revealed that more than half of the editing sites are edited by p150, with the other half edited by either p150 or p110 (26). ADAR1 and ADAR2 display different substrate selectivity, determined in part by their deaminase domains (44). However, analysis of recombinant chimeric proteins between ADAR1 and ADAR2 and between ADAR1 and PKR illustrates that substrate selectivity is determined by both the RNA binding domains (45) and the deaminase domain (44).

In mice, genetic ablation of *Adar1* gene function by disrupting expression of both p150 and p110 or by disrupting p150 expression alone, or by knock-in of the catalytically dead E861A mutant (corresponding to E912 in human ADAR1), results in embryonic lethality (31, 32, 46–48). This phenotype is largely rescued by a second concomitant knock-out of either the *Mda5* (*Ifih1*) sensor or the *Mavs* adapter (48–50), although the phenotypes of E861A knock-in *Adar1*, *Ifih1* deletion and the *Adar1*, *Mavs* double deletion are not equivalent (48–51).

## ADAR2

*ADAR2* expression is driven by multiple constitutive promoters and involves alternative splicing of transcripts. Major ADAR2 proteins in human cells predicted from cDNA sequence are 701 and 750 aa (17, 52). ADAR2 possesses two copies of the RNA-binding domain in the N-terminal region; the deaminase catalytic domain is present in the C-terminal region (Figure 2). ADAR2 does not have a Z domain. Unlike ADAR1, ADAR2 is not regulated by IFN (6, 7, 19, 53). ADAR2 is phosphorylated by protein kinase C at two sites between the dsRNA-binding domains, S211 and S216. Phosphorylation at these sites affects editing of microRNA (miR)-200 (54). ADAR2 is largely responsible for the highly site-selective A-to-I editing observed in mammalian cells (6, 25).

In mice, genetic ablation of *Adar2* does not result in embryonic lethality. However, *Adar2*-null mice have diminished survival. Postnatal death of mice lacking ADAR2 protein is largely rescued by the knock-in of the already-edited glutamate receptor (GluRB) Q/R-site (55). ADAR2 is required for normal physiology more broadly in mice (56). ADAR2 autoregulates its expression at the level of pre-mRNA splicing. Introns of the U2 type are flanked by 5’-GU–AG-3’ consensus splice-site sequences. Alternative 5’-AU or AA-3’ splice sites can be converted to the canonical splice-site sequence by A-to-I editing, as I is recognized as G by the spliceosome machinery. Such site-specific editing was demonstrated with ADAR2 pre-mRNA, where ADAR2 autoediting converts the AA-3’ sequence to AI-3’ (57). This type of editing affecting pre-mRNA splicing is rare, thus far observed for only a relatively limited number of cellular transcripts (58).

## ADAR3

Expression of ADAR3 is restricted to the nervous system; expression is high in the hippocampus and amygdala (59, 60). Human ADAR3 protein, predicted by cDNA sequence, is 739 aa and includes two copies of the R domain in the N-terminal region and a deaminase-like C-terminal domain (17, 59). While wild-type (WT) ADAR3 does not display detectable deaminase catalytic activity (4, 6, 61), mutation of human ADAR3 at five positions (A389V, V485I, E527Q, Q549R, and Q733D) generates an active enzyme (61). Binding of WT ADAR3 to dsRNA structures can affect the editing efficiency of ADAR2, illustrated by the ADAR3 inhibition of GluRB pre-mRNA editing in glioblastoma cells (62). In mice, genetic ablation of *Adar3* does not result in embryonic lethality. However, mice lacking Adar3 display deficits in hippocampus-dependent short- and long-term memory formation (60).

## EFFECTS OF ADARs AND A-TO-I DEAMINATION EDITING ON VIRUS-HOST INTERACTIONS

**Figure 4:**
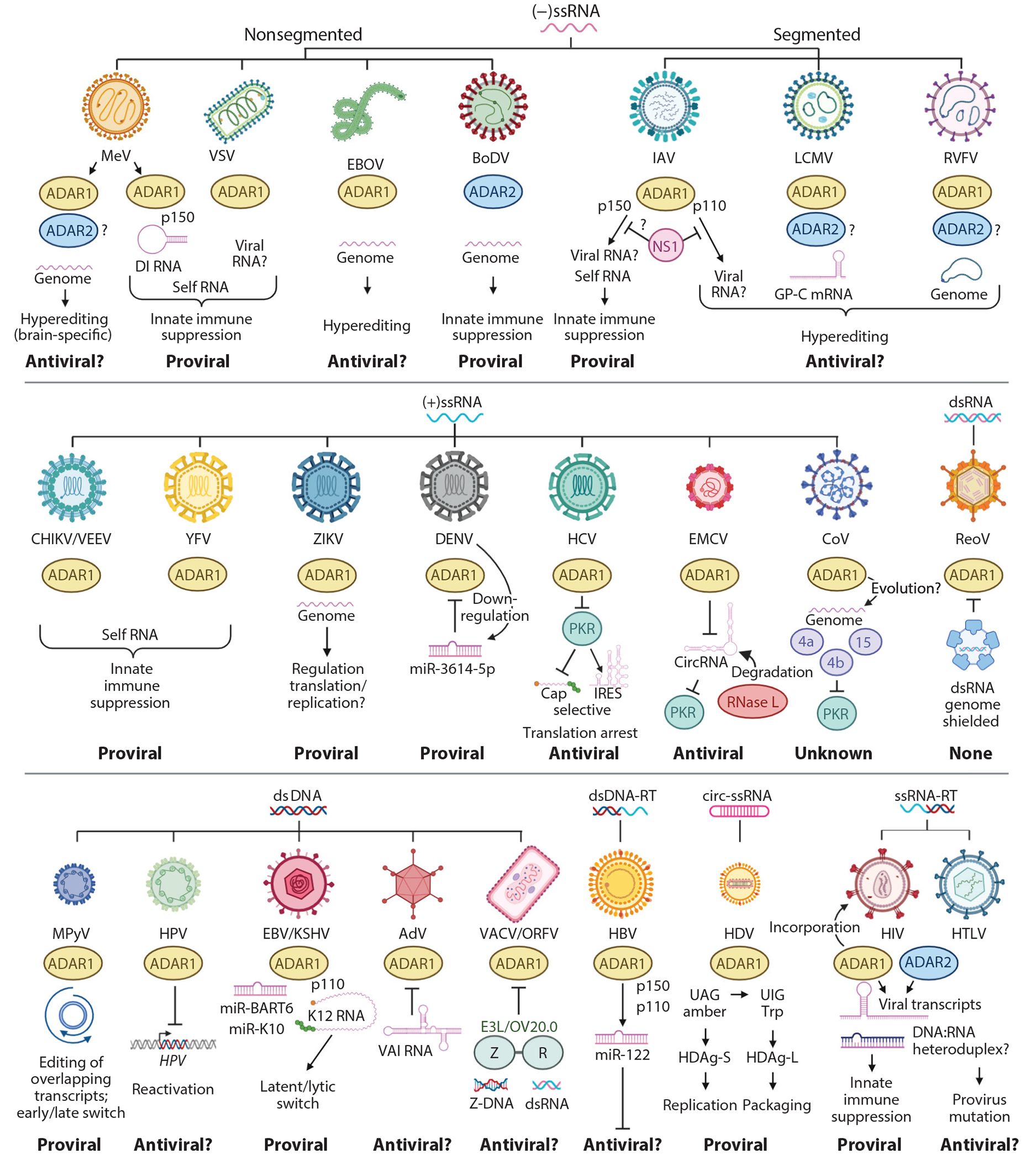
Effect of ADARs on virus replication and host responses to viral infection. ADARs are antiviral, proviral, or have no effect on virus infection, depending on the virus-host combination. Representative animal viruses are grouped by the biochemical nature of their genomes. The protein (ADAR1, *yellow*; ADAR2, *blue*) implicated in mediating the response, the viral or host RNA targeted by the ADAR protein, and the mechanism by which viral replication is affected are indicated. Abbreviations: ADARs, adenosine deaminases acting on RNA; AdV, adenovirus; BoDV, Borna disease virus; CHIKV, chikungunya virus; circ, circular; CoV, coronavirus; DENV, Dengue virus; DI, defective-interfering; ds, double-stranded; EBOV, Ebola virus; EBV, Epstein-Barr virus; EMCV, encephalomyocarditis virus; GP-C, glycoprotein precursor; HBV, hepatitis B virus; HCV, hepatitis C virus; HDV, hepatitis delta virus; HIV, human immunodeficiency virus; HPV, human papillomavirus; HTLV, human T cell leukemia virus; IAV, influenza A virus; IRES, internal ribosome entry site; KSHV, Kaposi sarcoma–associated herpesvirus; LCMV, lymphocytic choriomeningitis virus; MeV, measles virus; MPyV, murine polyomavirus; mRNA, messenger RNA; ORFV, Orf virus; PKR, protein kinase R; ReoV, reovirus; RT, reverse transcriptase; RVFV, Rift Valley fever virus; ss, single-stranded; VACV, vaccinia virus; VAI, adenovirus virus-associated RNA-I; VEEV, Venezuelan equine encephalitis virus; VSV, vesicular stomatitis virus; YFV, yellow fever virus; ZIKV, Zika virus. Figure adapted from images created with BioRender.com.

We now consider examples of virus infections affected by ADARs in either an antiviral or proviral manner as summarized by Figure 4.

### Measles Virus

Among negative-strand single-stranded (ss) RNA viruses (NSVs), much is known about A-to-I editing of measles virus (MeV) RNAs by ADARs (63). The viral genome, like that of other *Mononegavirales*, is transcribed and replicated by a virion-associated RNA polymerase. The MeV *P/V/C* gene-encoded accessory proteins C and V modulate innate immune responses, including synthesis of defective-interfering (DI) RNAs with ds structure that are PKR activators and ADAR substrates. Clustered A-to-I editing (hyperediting) of MeV RNA isolated from persistently infected brain tissue of subacute sclerosing panencephalitis (SSPE) patients (63) was discovered before ADAR enzymes were identified (2, 3). Editing disrupted viral protein production required for particle assembly, suggesting that editing may be linked directly to development of the often-fatal persistent viral infection. This process could be interpreted as the antiviral effect of ADAR. Because MeV exclusively replicates in the cytoplasm, editing likely is performed by cytoplasmic ADAR1-p150.

**Figure 5:**
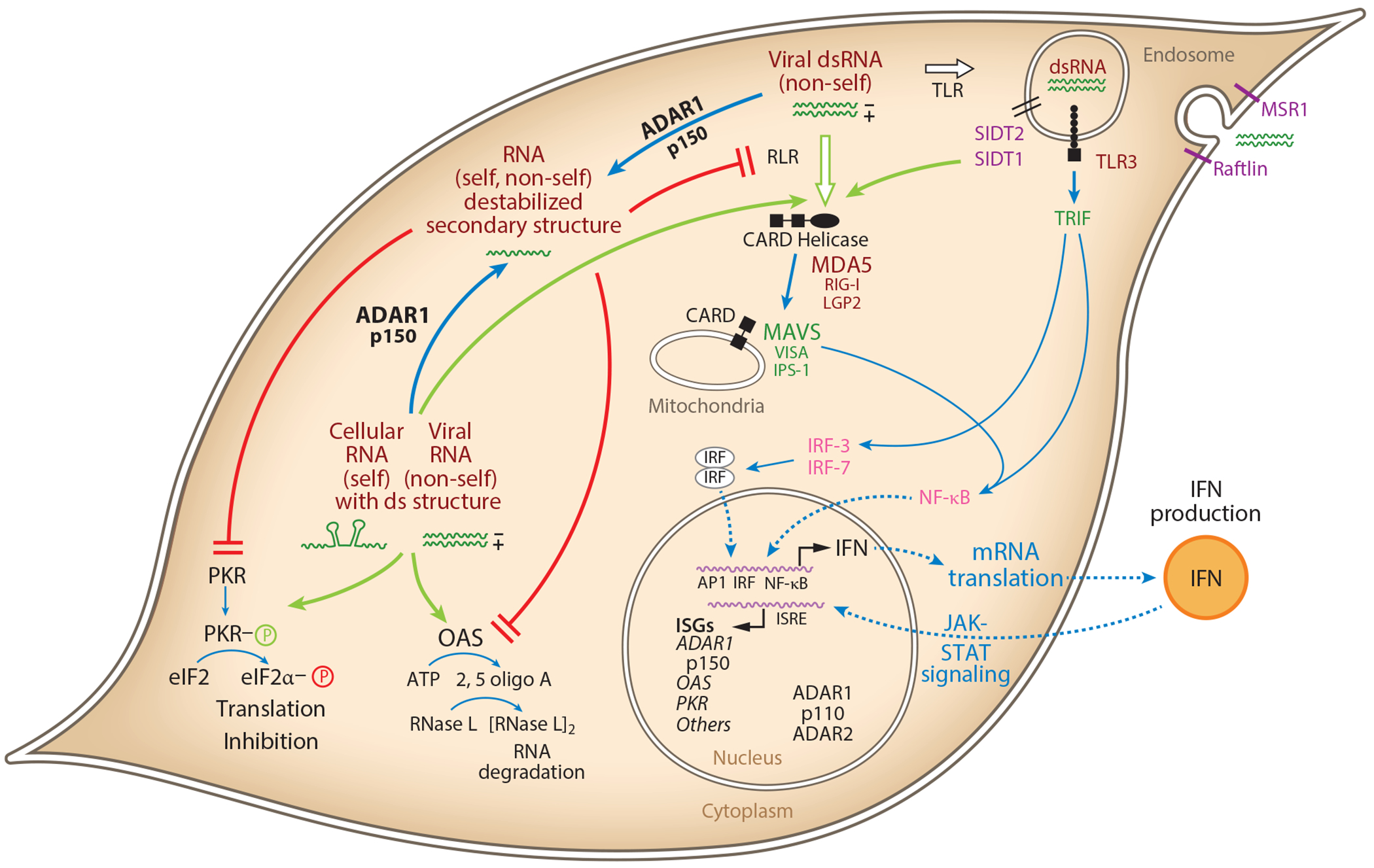
Model for ADAR1 as a suppressor of innate immune responses activated by dsRNA. Cytoplasmic RLRs MDA5 and RIG-I and endosome-associated TLR3 sense intracellular dsRNA to signal via MAVS and TRIF adapters the activation of IRF and NF-κB transcription factors that transcriptionally activate IFN expression. Extracellular viral dsRNAs may be transferred into uninfected bystander cells by the surface receptors Raftlin and MSR1 and internalized and transported to the cytoplasm by SIDT1/2 to trigger innate immune dsRNA sensors. Among the ISGs induced by IFN and JAK-STAT signaling are PKR and OAS, which, when activated by cytoplasmic dsRNA, lead to translation inhibition and RNA degradation. Under conditions of ADAR1-p150 deficiency, cytoplasmic viral (nonself) and cellular (self) RNAs with ds structure accumulate to a concentration surpassing the threshold required for activation of innate immune sensors including MDA5, PKR, and OAS, thereby leading to IFN production and action. In IFN-treated cells, cytoplasmic p150 is elevated, which leads to A-to-I editing and inactivation of cellular (self) dsRNAs that then fall below the threshold concentration necessary for innate immune sensor activation. Viral infection, however, may lead to sufficiently high concentrations of intracellular viral (nonself) dsRNAs that surpass the threshold for dsRNA sensor activation and trigger innate immune responses. Abbreviations: ADARs, adenosine deaminases acting on RNA; ds, double-stranded; IFN, interferon; IRF, interferon regulatory factor; ISRE, interferon-stimulated response element; JAK-STAT, Janus kinase-signal transducers and activators of transcription; mRNA, messenger RNA; OAS, oligoadenylate synthetase; PKR, protein kinase R; RLR, RIG-like receptor; SIDT, systemic RNA interference defective transmembrane; TLR, Toll-like receptor. Figure adapted from Reference 7.

Different from the rare SSPE disease, ADAR1 is generally proviral for acute MeV infection by counteracting PKR activation (64), stress granule formation (65), apoptosis (64), and type I IFN production (66). ADAR1-p150 hyperedits dsRNA-forming DI RNA of C protein–deficient MeV (43, 67, 68), which activates innate immune responses (69, 70). By altering DI RNA secondary structures, ADAR1 interferes with their recognition by dsRNA sensors including PKR (68). Likewise, ADAR1-p150 edits mRNA with *Alu*-duplex structures and prevents autoimmune reactions against self dsRNA (43, 53, 71, 72), suggesting that both viral and self RNA can trigger activation of PKR, MDA5, and oligoadenylate synthetase (OAS) (Figure 5), when the total amount of dsRNA exceeds the level that can be efficiently edited by ADAR1-p150 (43, 64–66, 68, 73–76). Notably, ADAR1 also appears to affect some adaptive T cell immune responses by a similar mechanism (77).

### Vesicular Stomatitis Virus

Similar to MeV, ADAR1 displays a proviral activity with vesicular stomatitis virus (VSV) (74). While ADAR1 deficiency does not affect virus yield per se (47, 74, 78), ADAR1-deficient cells display stronger PKR activation following treatment with type I IFN and thus mount stronger antiviral responses against VSV (74). IFN treatment may lead to expression of mRNA species with intrinsic dsRNA structures (43, 71, 72) that then activate PKR in the absence of ADAR1 (43, 53, 65, 66, 71). Alternatively, ADAR1 may directly block PKR activation through interactions of their R domains, as seen for some RNA-binding proteins (79, 80). Consistent with this observation, overexpression of ADAR1 enhances VSV replication in cells from WT mice but not from PKR-null animals (73).

### Ebola Virus

ADAR-like hypermutations are reported to accumulate in Ebola virus (EBOV) genomes in the glycoprotein gene when passaged in bat cells with high expression levels of ADAR1. However, such hypermutations are not observed following passaging of EBOV in human embryonic kidney (HEK)-293T cells with lower ADAR1 expression (81). Whether the effect is ADAR1 specific or also involves ADAR2 remains unclear. The observation is reminiscent of the hyperediting phenotype seen with MeV (63) and lymphocytic choriomeningitis virus (LCMV) (82) and suggests that ADAR1 has antiviral properties against EBOV and conceivably contributes to control of viral replication in bats.

### Borna Disease Virus

Borna disease virus (BoDV) is one of the few nonsegmented NSVs that replicates in the nucleus. While both ADAR1 and ADAR2 deficiencies reduce acute BoDV infection, establishment of persistent BoDV infection is reduced only in ADAR2-deficient cells (83). ADAR2-deficient cells exhibit an antiviral state characterized by upregulation of immune and inflammatory response genes, suggesting that ADAR2, like ADAR1, regulates autoimmune responses (43, 53, 71, 72). BoDV nuclear replication is reduced in ADAR2-deficient compared to ADAR2-sufficient cells (83). In addition, ADAR2 binds BoDV genomic RNA and introduces mutations, albeit at relatively low frequencies (83).

### Influenza A Virus

ADAR-editing has multifaceted effects on influenza A virus (IAV) infections. Some IAV strains induce upregulation of ADAR1 but not ADAR2, leading to increased A-to-I editing of cellular (84) and viral (85) RNAs. Moreover, ADAR-characteristic hypermutations are found in genomes of IAV vaccine preparations (86), However, ADAR1-p150 blocks sustained RIG-like receptor signaling (Figure 5); p150 is required for efficient IAV replication (87), in line with the reported proviral activity of ADAR1-p150 as a regulator of dsRNA-mediated innate immune responses (43, 64, 74). In contrast, reduced expression of ADAR1-p110 alone leads to an increase in viral replication, indicating a potential antiviral role of the p110 isoform (87). Thus, ADAR1-p110, which is nuclear, seemingly edits viral RNAs leading to an antiviral effect (84, 85), while cytoplasmic ADAR1-p150 limits dsRNA-mediated immune responses. Replicating IAV generates Z-RNAs that subsequently induce necroptosis via Z-DNA-binding protein 1, which possesses a Zα-domain like p150 (88). The IAV nonstructural protein 1 (NS1), a dsRNA-binding protein that is a potent PKR antagonist (15, 89), interacts with nuclear ADAR1 in a dsRNA-binding-independent manner through interactions with the R_I_ and R_II_ domains (90), suggesting that NS1 functions in reducing antiviral activities of p110 and thereby optimizes IAV replication.

### Arenaviruses and Bunyaviruses

ADAR-characteristic A-to-G and U-to-C hyperediting patterns are observed with LCMV of the *Arenaviridae* family and Rift Valley fever virus (RVFV) of the *Phenuiviridae* family. LCMV RNAs are synthesized by an ambisense replication strategy and can form an immunostimulatory dsRNA intermediate (91). ADAR-characteristic hypermutations are observed in the LCMV glycoprotein gene (82) and the RVFV L gene of a strain lacking functional IFN antagonist NSs (92). In both cases, editing is likely antiviral, as it impairs function of edited gene products. Notably, ADAR1-p150 expression is upregulated during LCMV infection (82, 91), and A-to-I-editing of RVFV RNA depends on a functional IFN system (92), which together suggest that the IFN-inducible ADAR1-p150 is responsible for the observed editing.

### Alphaviruses

A proviral activity of ADAR1 was identified for two alphaviruses, chikungunya virus (CHIKV) and Venezuelan equine encephalitis virus (VEEV). Replication of both viruses is enhanced in cells overexpressing ADAR1 (93). Curiously, this effect is observed in *Stat1*^−/−^ cells, initially suggesting that it was possibly independent of canonical type I IFN signaling (93). However, some interferon-stimulated genes (ISGs) including *ADAR1* p150 expression do not display a strict requirement for STAT1 (35). CHIKV replication is inhibited by the PKR-mediated stress responses (94). Whether ADAR1 protects CHIKV and VEEV from these responses that otherwise would induce shutdown of viral protein synthesis remains unknown.

### Flaviviruses

Flaviviruses, like alphaviruses, are zoonotic arthropod-borne viruses. Yellow fever virus (YFV) is a hepatotropic flavivirus. ADAR1 overexpression has a slight proviral effect on YFV replication (93), similar to CHIKV and VEEV. Zika virus (ZIKV) is a neurotropic flavivirus. Genomic analyses of multiple Old-World strains indicate an overrepresentation of G over A nucleotides at twofold-degenerated sites and synonymous codons (95, 96). These mutations coincide with regions forming dsRNA secondary structures and are consistent with A-to-I editing (95). ZIKV infections induce ADAR1 expression in neural stem cells and alter their proliferative capacity and neuronal differentiation (97), but it is unclear whether ADAR1 is directly responsible. In ADAR1-deficient cells, ZIKV replication is decreased and PKR activation increased (98). ADAR1-p110 expression rescues this phenotype (98). ZIKV-mediated induction of ADAR1 in neuronal cells may allow strong and neurotoxic viral replication and promote autoimmune responses such as Guillain-Barré syndrome (99). Dengue virus (DENV) induces ADAR1-p150 expression in monocyte-derived macrophages, resulting in downregulation of the antiviral miR-3614-5p (100). Notably, miR-3614-5p overexpression leads to reduction of DENV titers only in ADAR1-sufficient cells, not in ADAR1-deficient cells, indicating that miR-3614-5p function depends on ADAR1 (100).

### Hepatitis C Virus

Hepatitis C virus (HCV) is a blood-borne pathogen that replicates in the cytoplasm of hepatocytes, where it hijacks lipid droplets and reorganizes membrane structures for efficient viral replication (101) and innate immune evasion. Pegylated IFN-α therapy increases expression of ISGs including *ADAR1* (102). Increased A-to-I editing of ADAR1 targets, like the phosphodiesterase 8A (PDE8A) transcript (103), counteracts PKR activation (53, 64, 65), which for several viruses has a proviral effect (43, 66, 71, 74, 87). However, for HCV, increased PKR activity is beneficial for viral replication, as PKR-mediated translational arrest selectively blocks 5’-cap-dependent translation, while internal ribosome entry site–dependent translation initiation of HCV RNA is not affected (104). ADAR1 knock-down increases type I IFN responses, moderately stimulates HCV replication in Huh7 cells (105, 106), and leads to reduced sensitivity of HCV replication to IFN (106). In contrast, replication of an HCV replicon in ADAR1-deficient Huh7.5 cells, which express a nonfunctional RIG-I dsRNA sensor, is not altered relative to replication in WT cells (107). Taken together, ADAR1 appears to have antiviral activity against HCV that is achieved by limiting PKR-mediated translational arrest.

### Picornaviruses

Encephalomyocarditis virus (EMCV) is a member of the *Picornaviridae* family. Following infection with EMCV, viral circular RNAs (circRNAs) form that are 16–26-bp imperfect RNA duplexes; these circRNAs antagonize PKR activation. circRNAs are degraded by RNase L, a process required for PKR activation during early cellular innate immune responses (108). circRNAs are substrates for ADARs; knock-down of ADAR1 significantly upregulates circRNA expression (109).

### Coronaviruses

The *Coronaviridae* family includes the highly pathogenic Middle East respiratory syndrome (MERS) coronavirus (CoV); severe acute respiratory syndrome (SARS) CoV; and SARS-CoV-2, the causative agent of the pandemic outbreak of 2019–2020, causing coronavirus disease 2019 (COVID-19). CoVs produce dsRNA replication intermediates that can activate PKR and other dsRNA-mediated immune responses (110). Innate immune evasion occurs through CoV non-structural proteins NSP4a, a dsRNA-binding protein (111); MERS-NSP4b and mouse hepatitis virus (MHV)-NSP2, which are phosphodiesterases that antagonize RNase L activation (112, 113); and NSP15, an endoribonuclease required to evade MDA5, PKR, and OAS dsRNA sensors (114). ADAR deficiency leads to a dsRNA-activated OAS/RNase L response that is antagonized by the MHV-NSP2 (113). In genomes of SARS-CoV-2 isolates, nucleotide transitions characteristic of both ADAR and apolipoprotein B mRNA editing enzyme, catalytic polypeptide-like (APOBEC) activity are overrepresented (115, 116), but they do not display the hyperediting phenotype often seen with ADAR activity. Direct evidence for ADAR editing, such as comparative sequence analyses of SARS-CoV-2 propagated in the presence and absence of ADAR1, has not been reported.

### Reovirus

*Reoviridae* viruses have segmented dsRNA genomes and replicate in the cytoplasm. The synthesis of progeny genome dsRNA occurs within a core-like subvirion particle during virion morphogenesis (117), which efficiently shields viral dsRNA from some immune sensors including ADARs. No significant differences are observed in single-cycle reovirus (ReoV) yields in WT and ADAR1-p150-null mouse embryo fibroblasts (MEFs) (47), and *Adar1*^−/−^ cells lacking both p110 and p150 and *Adar2*^−/−^ MEFs (78). While infection of neonatal mice with the neurotropic ReoV Dearing strain results in significant ADAR1 induction in the brain, the induction results in few A-to-I(G) substitutions in virus-infected tissues as determined by high-throughput sequencing (27). Although ReoV expresses a dsRNA-binding protein (σ3), unlike vaccinia virus (VACV) E3L, σ3 has no inhibitory activity against ADAR1 editing of synthetic or natural dsRNA substrates (118). Taken together, these results suggest that ADARs surprisingly do not have a significant effect on ReoV infections, despite the large amounts of viral dsRNA present in reovirus-infected cells.

### Polyomavirus

Mouse polyomavirus (MPyV) has an ~5-kb circular dsDNA genome. DNA replication and subsequent virion assembly occur in the nucleus. Temporal regulation of bidirectional gene transcription by cellular RNA polymerase II occurs, giving rise to early and late viral transcripts made from opposite strands of the DNA genome. Late in the replication cycle, early mRNA is downregulated by late-strand RNA facilitating the switch from the production of T antigens to capsid proteins. An overlap between the 3’-ends of early and late transcripts forms a sense-antisense duplex, a viral dsRNA, which undergoes extensive A-to-I(G) substitutions as established by sequence analysis (119, 120).

Knock-down of Adar1 in 3T3 cells leads to a defect in the early-to-late switch and reduced expression of late transcripts (120). However, single-cycle yields and growth kinetics of MPyV are comparable in *Adar1*^−/−^ and *Adar2*^−/−^ cells. Although the expression of early proteins is greater in *Adar1*^−/−^ cells than in *Adar2*^−/−^ cells, the expression of late proteins is comparable in both mutant cell types and parallels comparable viral yields. However, the virus-induced cytopathic effect is greatly enhanced in *Adar1*^−/−^ cells relative to *Adar2*^−/−^ cells. Complementation with Adar1-p110 rescues cells from MPyV-induced cytotoxicity (78).

### Human Papillomavirus

Human papillomavirus (HPV) has an ~8-kb circular dsDNA genome that contains six early genes (E1, E2, E4, E5, E6, and E7) and two late genes (L1 and L2) transcribed by host RNA polymerase II in a temporally regulated manner. But unlike MPyV, HPV mRNAs are transcribed unidirectionally from one DNA strand. Ubiquitination of RIG-I mediated by the ubiquitin ligase tripartite motif-containing 25 (TRIM25) promotes dsRNA signaling to produce type I IFN; the HPV16 E6 oncoprotein targets TRIM25 to impair this signaling (121). ADAR1 regulates the expression of HPV proteins in keratinocyte cells (SiHa) in which the HPV16 genome is integrated (122, 123). ADAR1 knock-down upregulates HPV mRNA and protein (E1 and E7) expression. No A-to-I editing, however, is observed in HPV transcripts. It is postulated that downregulation of ADAR1 overcomes innate immune check-point blockade, thus facilitating enhanced viral expression (122). Furthermore, a genetic association between ADAR1 and HPV-induced dysplasia recurrence in HPV/human immunodeficiency virus (HIV) coinfected individuals has been observed using ADAR1 haplotype analysis, although the contribution of HIV infection was not excluded (122).

### Human Herpesviruses

*Herpesviridae* are large, enveloped viruses with linear dsDNA genomes ~125–250 kb in size. Viral DNA replication occurs in the nucleus, as does transcription by cellular RNA polymerase II. Epstein-Barr virus (EBV; human herpes virus 4) and Kaposi sarcoma–associated herpesvirus (KSHV; human herpes virus 8) transcripts include several viral long noncoding RNAs (vlncRNAs) and miRs. These RNAs modulate virus-host interactions and maintain viral latency or mediate the switch to the lytic cycle. Site-specific A-to-I(G) editing occurs of RNA transcripts encoded by these two human herpesviruses (124–126). Extensive A-to-I editing also is seen in malacoherpesvirus-infected mollusks, possibly by an ADAR1 homolog (127).

### Epstein-Barr Virus

Of the several EBV miR genes now known, primary transcripts of four miRs undergo site-specific A-to-I(G) editing: pri-miR-BART6, pri-miR-BART8, pri-miR-BART16, and pri-miR-BHRF1 (125). The frequency of editing by ADAR is greater with miR-BART6 relative to the others (125). miR-BART6 targets the DICER nuclease, and miR-BART6 binding to the DICER mRNA 3’ untranslated region (UTR) diminishes DICER expression, affecting mature miRs and influencing EBV latency. A-to-I(G) editing of the pri-miR-BART6 along with a deletion of three U residues causes the complete blockade of the Drosha cleavage step, eliminating the corresponding pre- and mature miR. However, editing alone is sufficient to inhibit the loading of miR-BART6-5P onto a functionally active miRNA-induced silencing complex (125). ADAR1 seems responsible for this editing based on enzyme expression levels. EBV-encoded miR-BART3 likewise undergoes A-to-I(G) editing (128), likely by ADAR1. EBV also expresses vlncRNAs during reactivation (126). One of these, oriPtLs, is A-to-I(G) hyperedited by ADAR1, consequently affecting viral lytic gene expression and viral DNA replication (126).

ADARs also edit cellular as well as viral miRs. A-to-I editing by ADAR1 and ADAR2 can affect the processing of primary miR transcripts (pri-miR) or the targeting of mature miRs (129, 130). It is estimated that ~20% of the pri-miRs are edited by ADARs (131). A-to-I editing of human miRs, likely by ADAR2, often is enriched in seed sequences and significantly hypoedited in glioblastoma (132).

### Kaposi Sarcoma–Associated Herpesvirus

KSHV causes two acquired immunodeficiency syndrome–associated malignancies, Kaposi sarcoma and primary effusion lymphoma. The viral K12 transcript is abundantly expressed and encodes three Kaposin proteins (A, B, and C). The region containing the ORF for Kaposin A also encodes miR-K10 and has tumorigenic potential (133, 134). Genome position 117990 falls within this region and is an A in the viral DNA but a G in RNA transcripts, indicating ADAR editing. This editing results in the substitution of a serine for a glycine in the Kaposin A protein. The change also affects miR-K10 with the potential to alter mRNA targeting. The biologic consequence of these changes is the elimination of transforming activity associated with the unedited A at 117990 and the restoration of lytic replication associated with a G(I) at this position (124). Purified ADAR1-p110 selectively edits the A at position 117990 of K12 RNA (124 ). ADAR1 isoforms increase throughout the viral lytic cycle progression, in parallel with an increase in the relative amount of edited Kaposin transcripts (135). Genetic recoding of mRNA decoding by ribosomes such as that observed with the Kaposin transcript is seen relatively infrequently with either viral or cellular mRNAs. Among the first and best-characterized examples of highly selective editing to change codons are the cellular pre-mRNAs for GluRB and 5HT2CR serotonin receptor pre-mRNAs and hepatitis delta virus (HDV) RNA (136–139).

### Adenovirus

Adenoviruses (AdVs) have linear dsDNA genomes ~30–36 kb in size. Viral DNA replication and transcription occur in the nucleus of infected cells. AdV mRNA transcripts are not known to be edited by ADARs. However, the small viral AdV virus-associated RNA-I (VAI) RNA transcribed by cellular RNA polymerase III is a potent inhibitor of ADAR1-mediated A-to-I(G) editing activity (140). Endogenous ADAR1 present in extracts from IFN-treated human U cells, or ADAR1-p150 or p110 ectopically expressed in COS cells, is inhibited by VAI RNA (140). VAI RNA is an antagonist of PKR and eukaryotic initiation factor-2α (eIF2α) phosphorylation; VAI RNA is required for efficient synthesis of both viral and cellular proteins at late time points after infection (141). Analysis of VAI RNA mutants suggests that the central domain of VAI is important in the antagonism of both ADAR1 and PKR but that the interactions of VAI with ADAR1 and PKR, while similar, are not equivalent with mutants that do not inhibit PKR but do inhibit ADAR1. VAI is not a detectable substrate of ADAR1 (140). The inhibitory activity of VAI is proposed to occur by its direct binding to the dsRNA-binding motifs of ADAR1, thus impeding its catalytic activity or otherwise blocking the binding of RNA substrates. It is not known whether VAI RNA inhibits the activity of ADAR2.

### Poxviruses

Poxviruses including VACV have linear dsDNA genomes ~200 kb in size. Unique among DNA viruses, replication of poxviruses occurs in the cytoplasm. Even though significant amounts of dsRNA are formed during VACV infection (14), none of the VACV transcripts are known to undergo A-to-I(G) editing. A viral dsRNA-binding protein, E3L, is a potent inhibitor of not only PKR (142, 143) but also ADAR1 (118). E3L has a single Z domain and a single R domain. One possible mechanism by which E3L impairs ADAR1 activity could be the sequestration of the RNA substrate (118). The carboxy-proximal R domain of E3L is essential for ADAR1 inhibitory activity. While deletion of the N-terminal Z domain has no effect on ADAR1 activity, disruption of the Z domain by double substitution of two conserved amino acids abolishes ADAR1 inhibition. Mutations decreasing Z-DNA binding of E3L also correlate with decreased viral pathogenicity. A chimeric VACV incorporating a related protein that does not bind Z-DNA is not pathogenic, but a mutation that allows Z-DNA binding generates a lethal virus. The capacity to bind the Z conformation is thus essential to E3L activity (144).

Orf virus (ORFV) is a poxvirus that infects sheep and goats. ORFV protein OV20.0 is an ortholog of the VACV E3L protein. OV20.0, like E3L, suppresses innate immune responses in part by inactivating PKR (145). ADAR1 plays a proviral role in ORFV infections by antagonizing PKR activation (145).

### Hepatitis B Virus

Hepatitis B virus (HBV) has an ~3.2-kb relaxed partially dsDNA genome that is replicated by reverse transcription of an RNA intermediate. Nuclear covalently closed circular viral DNA (cccDNA) produces the replicative intermediate pregenomic RNA (pgRNA) and viral mRNAs. HBc nucleocapsid protein, polymerase P protein, and pgRNA are assembled into a nucleocapsid, and the P converts pgRNA into relaxed circular DNA by its reverse transcriptase activity. Type I and III IFNs reduce nuclear cccDNA and HBV RNA levels in HBV-infected hepatocytes (146, 147). ADAR1 affects the replication of HBV, although the implicated mechanism differs between studies (147, 148).

ADAR1 inhibits HBV replication in hepatocyte Huh7 cells (147) by enhancing mirR-122 processing and hence increasing miR-122 levels that then downregulate HBV RNA. Both ADAR1-p110 and p150, but not ADAR2, reduce HBV RNA. A-to-I editing activity of ADAR1 is not required; WT ADAR1 and the E912Q mutant both decrease HBV RNA production. Induction of ADAR1 by IFN-α also reduces HBV RNA levels, whereas knock-down or knock-out of ADAR1 increases HBV RNA (147). In contrast to these findings implicating an miR-based antiviral mechanism for ADAR1 against HBV (147), ADAR1 also was reported to stimulate through its deaminase domain HBV DNA replication in hepatocellular carcinoma cells (148). Overexpression of ADAR1 promotes the replication of all four HBV genotypes.

The single nucleotide polymorphism rs4845384 in the 5’ UTR of the ADAR1 gene is associated with HBV clearance (149, 150). The allele rs4845384AA is associated with lower expression of ADAR1 and poorer response to IFN therapy compared with non-AA alleles. ADAR1 mRNA levels also are lower in persons with chronic hepatitis B compared with healthy individuals. Taken together, these findings suggest an antiviral role for ADAR1 in the context of HBV infection (147, 149, 150).

### Hepatitis Delta Virus

HDV is a defective human satellite virus requiring coinfection with HBV for replication (151). The genome of HDV is an ~1.7-kb circRNA that forms a rod-like imperfect dsRNA duplex transcribed apparently by a cellular DNA-dependent RNA polymerase by an unknown mechanism using an RNA template. HDV RNA encodes two forms of delta antigen: small delta antigen (HDAg-S) and large delta antigen (HDAg-L). Highly site-selective ADAR1-mediated editing of HDV RNA allows for HDAg-L synthesis: An amber UAG translation stop codon is converted to a UIG tryptophan codon. The A-to-I editing of HDV occurs on antigenome RNA in the nucleus primarily by ADAR1-p110 (152, 153). In the absence of editing, the HDAg-L protein is not synthesized, and HDV genome RNA is not packaged (154). Triggering of innate immune responses by HDV RNA occurs in part via the MDA5 sensor. However, innate immunity seems not to impair HDV while inhibiting HBV (151). Under normal physiologic conditions the amber/W-site editing is proviral, although increased expression of ADAR1-p150 by IFN treatment, or increased expression of either ADAR1 or ADAR2 by ectopic expression, causes an increase in HDV RNA editing and inhibition of HDV replication (155).

### Human Immunodeficiency Viruses

Multiple IFN-induced cellular proteins act as restriction factors against HIV type 1 (HIV-1) (89, 156), including the APOBEC3 family of deoxycytidine deaminases, tetherin that restricts virion release, TRIM5α that recognizes the capsid lattice and mediates disassembly, myxovirus B, a dynamin-like GTPase that prevents nuclear accumulation of HIV-1 provirus, and PKR. In contrast to these antiviral ISGs, ADAR1 is a proviral ISG and enhances HIV-1 gene expression and replication (157–159).

Ectopic overexpression of WT ADAR1 but not the catalytic mutant (Figure 2) increases expression of HIV-1 capsid CA protein and virion production, whereas ADAR1 knock-down decreases viral protein expression (157). In cells overexpressing ADAR1, A-to-G substitutions characteristic of ADAR editing are detected in the Tat and Rev encoding sequences in the vicinity of Rev response element and in the 5’ untranslated region but not around the *trans*-activation response element (157, 159). These results suggest that the proviral effect of ADAR1 is dependent at least in part on ADAR1 catalytic activity. However, other studies indicate that ADAR1 enhances HIV-1 replication by both editing-independent and editing-dependent means (157, 159). Overexpression of ADAR1-p150 enhances viral protein expression in a manner independent of deaminase activity but dependent on the dsRNA-binding domains. Although differences are observed between HEK-293T and COS7 cells in the apparent requirement for editing activity for the HIV-1 proviral effects, ADAR1 deficiency in cell lines and primary macrophages leads to decreased HIV-1 gene expression and virus replication and a type I IFN signature (157, 159, 160). ADAR2 also displays HIV-1 proviral activity and enhances HIV-1 replication (161), but whether the ADAR1 and ADAR2 activities toward HIV-1 are mechanistically similar or different is not yet clear.

One mechanism by which ADAR1 enhances the replication of multiple different RNA viruses, including HIV-1, is inhibition of PKR (7, 15, 162). ADAR1 interactions with PKR, and decreased PKR activation and eIF2α phosphorylation, occur under conditions of enhanced HIV-1 protein synthesis (158, 159, 163). ADAR1 also interacts with the HIV-1 p55 group-specific antigen, a structural protein (164). Endogenous ADAR1 present in T cells is incorporated into virions purified from HIV-1-infected T lymphocytes. The function of the incorporated ADAR1 has not yet been elucidated. It is possible that incorporation of ADAR1 into virions (164) restricts HIV, perhaps conceptually similar to the restriction mediated by APOBEC3 encapsidated into HIV virions that subsequently deaminates cytosines to uracils in viral cDNA (165).

Biochemical studies have shown DNA editing of DNA:RNA heteroduplex structures by ADARs at deoxyA-C mismatches in model substrates and by ADAR1 and ADAR2 with mutations (ADAR1 E1008Q and ADAR2 E488Q) in their base flipping region (166). It is unknown whether ADARs can edit 2’-deoxyadenosine in the DNA strand of retrovirus DNA:RNA heteroduplex structures. If this were to occur, ADARs potentially could negatively affect retrovirus replication by introduction of A-to-I(G) mutations into the proviral DNA. Examination of the ADAR1-ribonucleoprotein complex during HIV-1 replication led to the finding that ADAR1 also acts as a suppressor of LINE1 (long interspersed nuclear elements 1, or L1) retrotransposition (167). Although ADAR1 binds the basal L1 ribonucleoprotein complex, editing does not appear responsible for the inhibition of L1 retrotransposition (168).

### Other Retroviruses

Sequence changes characteristic of hyperediting by ADARs were reported in the mid-1990s for two retroviruses other than HIV-1: Rous-associated virus and avian leukosis virus (169, 170). The A-to-G mutations were found in the U3 long terminal repeat region and an inverted repeat region where nearly 50% of the adenosines were substituted with guanosines. It remains unknown which of the ADARs is responsible for these A-to-G mutations. Extensive A-to-I(G) hyperediting also is observed with human T cell leukemia virus type 2 (HTLV-2) and simian T cell leukemia virus type 3 and is attributed to ADAR1 (171). ADAR1 enhances HTLV-1 and HTLV-2 infection of T lymphocytes, a proviral effect that is independent of ADAR1 catalytic activity and again thought to occur by PKR antagonism (172).

## MODULATORS OF ADARs

Studies of poxvirus E3L and AdV VAI gene products first demonstrated that ADAR editing activity could be inhibited by a heterologous RNA-binding protein or structured RNA. However, the full significance of the inhibition by E3L and VAI is not yet established, as neither VACV nor AdV transcript editing has been described. Conceivably, the physiologic effects in part are independent of editing activity. Proteins and RNAs from many viruses including the NS1 protein of influenza virus affect PKR activity (15, 80, 89, 173); conceivably, some of them also affect ADAR activity, either RNA binding or enzymic or both. In contrast to VACV E3L that antagonizes ADAR1, the influenza virus NS1 and DENV NS3 proteins interact with ADAR1 in a dsRNA-binding independent manner to enhance editing activity (90). ADAR1 and PKR are recognized by nonoverlapping domains of NS1 (90).

Cellular antagonists of ADAR editing activity likewise are known. For example, binding of ADAR3 to the substrate GluRB pre-mRNA impairs ADAR2 editing of the Q/R site (62). ADAR1 and ADAR2 function as dimers; hence, they are likely subject to dominant-negative effects (174, 175). From a screen for repressors of editing, RNA binding proteins SRSF9 and RPS14 were identified that repress ADAR2 editing (176, 177). Multiple proteins were identified as regulators of ADAR1 editing through the use of RNA-sequencing and global protein-RNA binding data (178). These include TDP-43, Drosha, nuclear factor 45/90, and Ro60 that affect editing using multiple mechanisms including regulation of *ADAR1* expression, interaction with ADAR1 protein, and binding to *Alu* RNA elements. DExH-box helicase 9 binds to inverted repeat *Alu* elements. ADAR1-p150, but not p110, is an RNA-independent interaction partner (179). BioID followed by mass spectrometry identified regulatory proteins of ADAR1 and ADAR2, including four dimerization zinc finger domain–containing proteins (180, 181). ADAR1-p110 competitively inhibits binding of Staufen1, a cellular dsRNA-binding protein, to 3’-UTR dsRNAs and antagonizes Staufen1-mediated mRNA decay (41). Whether cellular antagonists or agonists of ADARs are affected by viral infection has not been reported.

## VIRAL DOUBLE-STRANDED RNA IN UNINFECTED BYSTANDER CELLS

dsRNA structures are produced in virus-infected cells by multiple mechanisms depending on the specific virus and its genome expression strategy. For example, ssRNA viruses produce dsRNA as part of the replicative intermediate or DI RNAs during RNA replication (43, 65, 74, 182), dsDNA viruses produce opposing transcripts that overlap in part (119, 120), and both viral and cellular transcripts can include regions with inverted complementary sequence (5, 53). These intracellular viral dsRNAs can activate cytoplasmic innate dsRNA sensors, and in some cases, they are suppressed by ADAR1 (Figure 5). Infected cells also can transfer extracellular viral dsRNAs, as exemplified by EMCV and herpes simplex virus type 1infections (183), to uninfected bystander cells by cell-surface receptors together with systemic RNA interference defective transmembrane (SIDT) proteins. SIDT1/2 transport dsRNA from endocytic compartments into the cytoplasm, thereby triggering dsRNA sensors (183, 184). SIDT2 loss results in impaired RIG-receptor signaling and impaired IFN production (183). Itis unknown whether such extracellular dsRNA entering into bystander cells also is subject to suppression by ADAR-mediated deamination as with dsRNA synthesized and acting within virus-infected cells.

### SUMMARY: CHALLENGES AND OPPORTUNITIES

The year 2020 brought a multitude of challenges and revealed opportunities, for science and for society. It was not one of the best years. There was considerable hardship. Lives were upended by and lost due to the COVID-19 pandemic, social injustices, and economic hardships. But with resilience, focus, and dedication, and by working together and with the power of science, we hope to soon benefit from the development of effective vaccines and immunization against SARS-CoV-2. For ADARs and A-to-I editing, there too are challenges and opportunities to consider as we move forward. While much has been learned about the biochemical mechanisms by which ADARs mediate biologic change, much remains to be discovered about their functions during viral infections.

For ADAR1, a major and potentially sole essential role of p150 is to suppress auto-triggering of innate immune IFN responses mediated by cytoplasmic dsRNA sensors including MDA5, PKR, and OAS. But whether these suppressive responses are the sole result of creating I-U mismatches that destabilize dsRNA structures, or whether I acts in another manner as a tag to signal responses, remains an open question. The finding that ADAR1 plays such a key role in regulating both autotriggered innate responses in uninfected cells and responses activated by viral dsRNA in infected cells presents opportunities to control the innate response. Perhaps development of pharmacologic agents that impair ADAR1 provides an opportunity to modulate the robustness of innate immune responses. Advances in sequencing strategies together with the use of model organisms and cell-culture systems possessing deficiencies in ADARs continue to provide insights into the functional importance of ADARs under a variety of conditions, including viral infection and IFN treatment. It will be important to gain further understanding of the roles that individual ADARs play and the extent, if any, to which their activities are redundant. Also, further understanding is needed about how the expression of individual ADARs is regulated, in addition to transcriptional activation of ADAR-p150 by IFN and enzymic activity by post-translational modifications including sumoylation and phosphorylation. The biochemical basis for the substrate selectivity of ADAR1 and ADAR2 enzymes and the functions of the repeated nucleic acid binding R and Z domains are not yet fully delineated. Nor is it fully resolved how, and under what conditions including infection, ADARs are regulated by interactions with cellular and viral dsRNA-binding proteins; by nucleic acids, as illustrated by the viral VAI RNA; and by other modifications of A such as reversible N6 methylation and how this may influence the action of ADARs whose C6 deamination is irreversible. Much has been learned about the roles that ADARs play in biology. But much remains to be learned about their importance, both as nucleic acid binding proteins and as enzymes that functionally substitute a G for an A.

## References

[1] Bass BL, Nishikura K, Keller W, Seeburg PH, Emeson RB, 1997. A standardized nomenclature for adenosine deaminases that act on RNA. RNA 3:947–49 9292492 PMC1369539

[2] Bass BL, Weintraub H. 1987. A developmentally regulated activity that unwinds RNA duplexes. Cell 48:607–13 2434241 10.1016/0092-8674(87)90239-x

[3] Rebagliati MR, Melton DA. 1987. Antisense RNA injections in fertilized frog eggs reveal an RNA duplex unwinding activity. Cell 48:599–605 2434240 10.1016/0092-8674(87)90238-8

[4] Nishikura K. 2016. A-to-I editing of coding and non-coding RNAs by ADARs. Nat. Rev. Mol. Cell Biol 17:83–96 26648264 10.1038/nrm.2015.4 PMC4824625

[5] Walkley CR, Li JB. 2017. Rewriting the transcriptome: adenosine-to-inosine RNA editing by ADARs. Genome Biol. 18:205 29084589 10.1186/s13059-017-1347-3 PMC5663115

[6] Eisenberg E, Levanon EY. 2018. A-to-I RNA editing—immune protector and transcriptome diversifier. Nat. Rev. Genet 19:473–90 29692414 10.1038/s41576-018-0006-1

[7] Samuel CE. 2019. Adenosine deaminase acting on RNA (ADAR1), a suppressor of double-stranded RNA-triggered innate immune responses. J. Biol. Chem 294:1710–20 30710018 10.1074/jbc.TM118.004166 PMC6364763

[8] Reich DP, Bass BL. 2019. Mapping the dsRNA world. Cold Spring Harb. Perspect. Biol 11:a035352 30824577 10.1101/cshperspect.a035352 PMC6396333

[9] Strobel SA, Cech TR, Usman N, Beigelman L. 1994. The 2,6-diaminopurine riboside·5-methylisocytidine wobble base pair: an isoenergetic substitution for the study of G·U pairs in RNA. Biochemistry 33:13824–35 7524665 10.1021/bi00250a037

[10] Hershfield MS. 2003. Genotype is an important determinant of phenotype in adenosine deaminase deficiency. Curr. Opin. Immunol 15:571–77 14499267 10.1016/s0952-7915(03)00104-3

[11] Rice GI, Kasher PR, Forte GM, Mannion NM, Greenwood SM, 2012. Mutations in *ADAR1* cause Aicardi-Goutières syndrome associated with a type I interferon signature. Nat. Genet 44:1243–48 23001123 10.1038/ng.2414 PMC4154508

[12] Herbert A. 2020. Mendelian disease caused by variants affecting recognition of Z-DNA and Z-RNA by the Zα domain of the double-stranded RNA editing enzyme ADAR. Eur. J. Hum. Genet 28:114–17 31320745 10.1038/s41431-019-0458-6 PMC6906422

[13] Miyamura Y, Suzuki T, Kono M, Inagaki K, Ito S, 2003. Mutations of the RNA-specific adenosine deaminase gene (DSRAD) are involved in dyschromatosis symmetrica hereditaria. Am. J. Hum. Genet 73:693–99 12916015 10.1086/378209 PMC1180697

[14] Colby C, Duesberg PH. 1969. Double-stranded RNA in vaccinia virus infected cells. Nature 222:940–44 5789322 10.1038/222940a0

[15] Samuel CE. 2011. Adenosine deaminases acting on RNA (ADARs) are both antiviral and proviral. Virology 411:180–93 21211811 10.1016/j.virol.2010.12.004 PMC3057271

[16] Hur S. 2019. Double-stranded RNA sensors and modulators in innate immunity. Annu. Rev. Immunol 37:349–75 30673536 10.1146/annurev-immunol-042718-041356 PMC7136661

[17] George CX, Gan Z, Liu Y, Samuel CE. 2011. Adenosine deaminases acting on RNA, RNA editing, and interferon action. J. Interferon Cytokine Res 31:99–117 21182352 10.1089/jir.2010.0097 PMC3034097

[18] Tomaselli S, Galeano F, Locatelli F, Gallo A. 2015. ADARs and the balance game between virus infection and innate immune cell response. Curr. Issues Mol. Biol 17:37–51 25502818

[19] Lamers MM, van den Hoogen BG, Haagmans BL. 2019. ADAR1: “editor-in-chief” of cytoplasmic innate immunity. Front. Immunol 10:1763 31404141 10.3389/fimmu.2019.01763 PMC6669771

[20] Patterson JB, Samuel CE. 1995. Expression and regulation by interferon of a double-stranded-RNA-specific adenosine deaminase from human cells: evidence for two forms of the deaminase. Mol. Cell. Biol 15:5376–88 7565688 10.1128/mcb.15.10.5376 PMC230787

[21] Weier HU, George CX, Greulich KM, Samuel CE. 1995. The interferon-inducible, double-stranded RNA-specific adenosine deaminase gene (DSRAD) maps to human chromosome 1q21.1–21.2. Genomics 30:372–75 8586444 10.1006/geno.1995.0034

[22] Kim U, Wang Y, Sanford T, Zeng Y, Nishikura K. 1994. Molecular cloning of cDNA for double-stranded RNA adenosine deaminase, a candidate enzyme for nuclear RNA editing. PNAS 91:11457–61 7972084 10.1073/pnas.91.24.11457 PMC45250

[23] O’Connell MA, Krause S, Higuchi M, Hsuan JJ, Totty NF, 1995. Cloning of cDNAs encoding mammalian double-stranded RNA-specific adenosine deaminase. Mol. Cell. Biol 15:1389–97 7862132 10.1128/mcb.15.3.1389 PMC230363

[24] George CX, Wagner MV, Samuel CE. 2005. Expression of interferon-inducible RNA adenosine deaminase ADAR1 during pathogen infection and mouse embryo development involves tissue-selective promoter utilization and alternative splicing. J. Biol. Chem 280:15020–28 15677478 10.1074/jbc.M500476200

[25] Tan MH, Li Q, Shanmugam R, Piskol R, Kohler J, 2017. Dynamic landscape and regulation of RNA editing in mammals. Nature 550:249–54 29022589 10.1038/nature24041 PMC5723435

[26] Sun T, Yu Y, Wu X, Acevedo A, Luo J-D, 2021. Decoupling expression and editing preferences of ADAR1 p150 and p110 isoforms. PNAS 118:e2021757118 33723056 10.1073/pnas.2021757118 PMC8000508

[27] Hood JL, Morabito MV, Martinez CR 3rd, Gilbert JA, Ferrick EA, 2014. Reovirus-mediated induction of ADAR1 (p150) minimally alters RNA editing patterns in discrete brain regions. Mol. Cell. Neurosci 61:97–109 24906008 10.1016/j.mcn.2014.06.001 PMC4134954

[28] George CX, Samuel CE. 1999. Human RNA-specific adenosine deaminase *ADAR1* transcripts possess alternative exon 1 structures that initiate from different promoters, one constitutively active and the other interferon inducible. PNAS 96:4621–26 10200312 10.1073/pnas.96.8.4621 PMC16382

[29] George CX, Samuel CE. 1999. Characterization of the 5’-flanking region of the human RNA-specific adenosine deaminase *ADAR*1 gene and identification of an interferon-inducible *ADAR*1 promoter. Gene 229:203–13 10095120 10.1016/s0378-1119(99)00017-7

[30] Kawakubo K, Samuel CE. 2000. Human RNA-specific adenosine deaminase (*ADAR1*) gene specifies transcripts that initiate from a constitutively active alternative promoter. Gene 258:165–72 11111054 10.1016/s0378-1119(00)00368-1

[31] Hartner JC, Schmittwolf C, Kispert A, Müller AM, Higuchi M, Seeburg PH. 2004. Liver disintegration in the mouse embryo caused by deficiency in the RNA-editing enzyme ADAR1. J. Biol. Chem 279:4894–902 14615479 10.1074/jbc.M311347200

[32] Wang Q, Miyakoda M, Yang W, Khillan J, Stachura DL, 2004. Stress-induced apoptosis associated with null mutation of *ADAR1* RNA editing deaminase gene. J. Biol. Chem 279:4952–61 14613934 10.1074/jbc.M310162200

[33] Stark GR, Darnell JE Jr. 2012. The JAK-STAT pathway at twenty. Immunity 36:503–14 22520844 10.1016/j.immuni.2012.03.013 PMC3909993

[34] George CX, Das S, Samuel CE. 2008. Organization of the mouse RNA-specific adenosine deaminase *Adar1* gene 5’-region and demonstration of STAT1-independent, STAT2-dependent transcriptional activation by interferon. Virology 380:338–43 18774582 10.1016/j.virol.2008.07.029 PMC2628478

[35] George CX, Samuel CE. 2015. STAT2-dependent induction of RNA adenosine deaminase ADAR1 by type I interferon differs between mouse and human cells in the requirement for STAT1. Virology 485:363–70 26335850 10.1016/j.virol.2015.08.001 PMC4619148

[36] Herbert A. 2019. Z-DNA and Z-RNA in human disease. Commun. Biol 2:7 30729177 10.1038/s42003-018-0237-x PMC6323056

[37] Nichols PJ, Bevers S, Henen M, Kieft JS, Vicens Q, Vogeli B. 2021. Recognition of non-CpG repeats in Alu and ribosomal RNAs by the Z-RNA binding domain of ADAR1 induces A-Z junctions. Nat. Commun 12:793 33542240 10.1038/s41467-021-21039-0 PMC7862695

[38] Liu Y, Samuel CE. 1996. Mechanism of interferon action: functionally distinct RNA-binding and catalytic domains in the interferon-inducible, double-stranded RNA-specific adenosine deaminase. J. Virol 70:1961–68 8627722 10.1128/jvi.70.3.1961-1968.1996 PMC190025

[39] Desterro JM, Keegan LP, Jaffray E, Hay RT, O’Connell MA, Carmo-Fonseca M. 2005. SUMO-1 modification alters ADAR1 editing activity. Mol. Biol. Cell 16:5115–26 16120648 10.1091/mbc.E05-06-0536 PMC1266412

[40] Bavelloni A, Focaccia E, Piazzi M, Raffini M, Cesarini V, 2019. AKT-dependent phosphorylation of the adenosine deaminases ADAR-1 and -2 inhibits deaminase activity. FASEB J. 33:9044–61 31095429 10.1096/fj.201800490RR

[41] Sakurai M, Shiromoto Y, Ota H, Song C, Kossenkov AV, 2017. ADAR1 controls apoptosis of stressed cells by inhibiting Staufen1-mediated mRNA decay. Nat. Struct. Mol. Biol 24:534–43 28436945 10.1038/nsmb.3403 PMC5461201

[42] Schade M, Turner CJ, Kühne R, Schmieder P, Lowenhaupt K, 1999. The solution structure of the Zα domain of the human RNA editing enzyme ADAR1 reveals a prepositioned binding surface for Z-DNA. PNAS 96:12465–70 10535945 10.1073/pnas.96.22.12465 PMC22950

[43] Pfaller CK, Donohue RC, Nersisyan S, Brodsky L, Cattaneo R. 2018. Extensive editing of cellular and viral double-stranded RNA structures accounts for innate immunity suppression and the proviral activity of ADAR1^p150^. PLOS Biol. 16:e2006577 30496178 10.1371/journal.pbio.2006577 PMC6264153

[44] Wang Y, Park S, Beal PA. 2018. Selective recognition of RNA substrates by ADAR deaminase domains. Biochemistry 57:1640–51 29457714 10.1021/acs.biochem.7b01100 PMC5898644

[45] Liu Y, Lei M, Samuel CE. 2000. Chimeric double-stranded RNA-specific adenosine deaminase ADAR1 proteins reveal functional selectivity of double-stranded RNA-binding domains from ADAR1 and protein kinase PKR. PNAS 97:12541–46 11070079 10.1073/pnas.97.23.12541 PMC18800

[46] Hartner JC, Walkley CR, Lu J, Orkin SH. 2009. ADAR1 is essential for the maintenance of hematopoiesis and suppression of interferon signaling. Nat. Immunol 10:109–15 19060901 10.1038/ni.1680 PMC2701568

[47] Ward SV, George CX, Welch MJ, Liou LY, Hahm B, 2011. RNA editing enzyme adenosine deaminase is a restriction factor for controlling measles virus replication that also is required for embryogenesis. PNAS 108:331–36 21173229 10.1073/pnas.1017241108 PMC3017198

[48] Liddicoat BJ, Piskol R, Chalk AM, Ramaswami G, Higuchi M, 2015. RNA editing by ADAR1 prevents MDA5 sensing of endogenous dsRNA as nonself. Science 349:1115–20 26275108 10.1126/science.aac7049 PMC5444807

[49] Mannion NM, Greenwood SM, Young R, Cox S, Brindle J, 2014. The RNA-editing enzyme ADAR1 controls innate immune responses to RNA. Cell Rep. 9:1482–94 25456137 10.1016/j.celrep.2014.10.041 PMC4542304

[50] Pestal K, Funk CC, Snyder JM, Price ND, Treuting PM, Stetson DB. 2015. Isoforms of RNA-editing enzyme ADAR1 independently control nucleic acid sensor MDA5-driven autoimmunity and multi-organ development. Immunity 43:933–44 26588779 10.1016/j.immuni.2015.11.001 PMC4654992

[51] Bajad P, Ebner F, Amman F, Szabó B, Kapoor U, 2020. An internal deletion of ADAR rescued by MAVS deficiency leads to a minute phenotype. Nucleic Acids Res. 48:3286–303 31956894 10.1093/nar/gkaa025 PMC7102943

[52] Gerber A, O’Connell MA, Keller W. 1997. Two forms of human double-stranded RNA-specific editase 1 (hRED1) generated by the insertion of an Alu cassette. RNA 3:453–63 9149227 PMC1369496

[53] George CX, Ramaswami G, Li JB, Samuel CE. 2016. Editing of cellular self-RNAs by adenosine deaminase ADAR1 suppresses innate immune stress responses. J. Biol. Chem 291:6158–68 26817845 10.1074/jbc.M115.709014 PMC4813567

[54] Shelton PM, Duran A, Nakanishi Y, Reina-Campos M, Kasashima H, 2018. The secretion of miR-200s by a PKCζ/ADAR2 signaling axis promotes liver metastasis in colorectal cancer. Cell Rep. 23:1178–91 29694894 10.1016/j.celrep.2018.03.118 PMC5958623

[55] Higuchi M, Maas S, Single FN, Hartner J, Rozov A, 2000. Point mutation in an AMPA receptor gene rescues lethality in mice deficient in the RNA-editing enzyme ADAR2. Nature 406:78–81 10894545 10.1038/35017558

[56] Horsch M, Seeburg PH, Adler T, Aguilar-Pimentel JA, Becker L, 2011. Requirement of the RNA-editing enzyme ADAR2 for normal physiology in mice. J. Biol. Chem 286:18614–22 21467037 10.1074/jbc.M110.200881 PMC3099677

[57] Dawson TR, Sansam CL, Emeson RB. 2004. Structure and sequence determinants required for the RNA editing of ADAR2 substrates. J. Biol. Chem 279:4941–51 14660658 10.1074/jbc.M310068200

[58] Kapoor U, Licht K, Amman F, Jakobi T, Martin D, 2020. ADAR-deficiency perturbs the global splicing landscape in mouse tissues. Genome Res. 30:1107–18 32727871 10.1101/gr.256933.119 PMC7462079

[59] Chen CX, Cho DS, Wang Q, Lai F, Carter KC, Nishikura K. 2000. A third member of the RNA-specific adenosine deaminase gene family, ADAR3, contains both single- and double-stranded RNA binding domains. RNA 6:755–67 10836796 10.1017/s1355838200000170 PMC1369955

[60] Mladenova D, Barry G, Konen LM, Pineda SS, Guennewig B, 2018. Adar3 is involved in learning and memory in mice. Front. Neurosci 12:243 29719497 10.3389/fnins.2018.00243 PMC5914295

[61] Wang Y, Chung DH, Monteleone LR, Li J, Chiang Y, 2019. RNA binding candidates for human ADAR3 from substrates of a gain of function mutant expressed in neuronal cells. Nucleic Acids Res. 47:10801–14 31552420 10.1093/nar/gkz815 PMC6846710

[62] Oakes E, Anderson A, Cohen-Gadol A, Hundley HA. 2017. Adenosine deaminase that acts on RNA 3 (ADAR3) binding to glutamate receptor subunit B pre-mRNA inhibits RNA editing in glioblastoma. J. Biol. Chem 292:4326–35 28167531 10.1074/jbc.M117.779868 PMC5354488

[63] Cattaneo R, Schmid A, Eschle D, Baczko K, ter Meulen V, Billeter MA. 1988. Biased hypermutation and other genetic changes in defective measles viruses in human brain infections. Cell 55:255–65 3167982 10.1016/0092-8674(88)90048-7 PMC7126660

[64] Toth AM, Li Z, Cattaneo R, Samuel CE. 2009. RNA-specific adenosine deaminase ADAR1 suppresses measles virus-induced apoptosis and activation of protein kinase PKR. J. Biol. Chem 284:29350–56 19710021 10.1074/jbc.M109.045146 PMC2785566

[65] Okonski KM, Samuel CE. 2013. Stress granule formation induced by measles virus is protein kinase PKR dependent and impaired by RNA adenosine deaminase ADAR1. J. Virol 87:756–66 23115276 10.1128/JVI.02270-12 PMC3554044

[66] Li Z, Okonski KM, Samuel CE. 2012. Adenosine deaminase acting on RNA 1 (ADAR1) suppresses the induction of interferon by measles virus. J. Virol 86:3787–94 22278222 10.1128/JVI.06307-11 PMC3302501

[67] Pfaller CK, Radeke MJ, Cattaneo R, Samuel CE. 2014. Measles virus C protein impairs production of defective copyback double-stranded viral RNA and activation of protein kinase R. J. Virol 88:456–68 24155404 10.1128/JVI.02572-13 PMC3911759

[68] Pfaller CK, Mastorakos GM, Matchett WE, Ma X, Samuel CE, Cattaneo R. 2015. Measles virus defective interfering RNAs are generated frequently and early in the absence of C protein and can be destabilized by adenosine deaminase acting on RNA-1-like hypermutations. J. Virol 89:7735–47 25972541 10.1128/JVI.01017-15 PMC4505647

[69] Toth AM, Devaux P, Cattaneo R, Samuel CE. 2009. Protein kinase PKR mediates the apoptosis induction and growth restriction phenotypes of C protein-deficient measles virus. J. Virol 83:961–68 19004947 10.1128/JVI.01669-08 PMC2612345

[70] McAllister CS, Toth AM, Zhang P, Devaux P, Cattaneo R, Samuel CE. 2010. Mechanisms of protein kinase PKR-mediated amplification of beta interferon induction by C protein-deficient measles virus. J. Virol 84:380–86 19846517 10.1128/JVI.02630-08 PMC2798421

[71] Chung H, Calis JJA, Wu X, Sun T, Yu Y, 2018. Human ADAR1 prevents endogenous RNA from triggering translational shutdown. Cell 172:811–24.e14 29395325 10.1016/j.cell.2017.12.038 PMC5831367

[72] Ahmad S, Mu X, Yang F, Greenwald E, Park JW, 2018. Breaching self-tolerance to Alu duplex RNA underlies MDA5-mediated inflammation. Cell 172:797–810.e13 29395326 10.1016/j.cell.2017.12.016 PMC5807104

[73] Nie Y, Hammond GL, Yang JH. 2007. Double-stranded RNA deaminase ADAR1 increases host susceptibility to virus infection. J. Virol 81:917–23 17079286 10.1128/JVI.01527-06 PMC1797455

[74] Li Z, Wolff KC, Samuel CE. 2010. RNA adenosine deaminase ADAR1 deficiency leads to increased activation of protein kinase PKR and reduced vesicular stomatitis virus growth following interferon treatment. Virology 396:316–22 19913273 10.1016/j.virol.2009.10.026 PMC2789878

[75] Yang S, Deng P, Zhu Z, Zhu J, Wang G, 2014. Adenosine deaminase acting on RNA1 limits RIG-I RNA detection and suppresses IFN production responding to viral and endogenous RNAs. J. Immunol 193:3436–45 25172485 10.4049/jimmunol.1401136 PMC4169998

[76] Li Y, Banerjee S, Goldstein SA, Dong B, Gaughan C, 2017. Ribonuclease L mediates the cell-lethal phenotype of double-stranded RNA editing enzyme ADAR1 deficiency in a human cell line. eLife 6:e25687 28362255 10.7554/eLife.25687 PMC5404912

[77] Nakahama T, Kato Y, Kim JI, Vongpipatana T, Suzuki Y, 2018. ADAR1-mediated RNA editing is required for thymic self-tolerance and inhibition of autoimmunity. EMBO Rep. 19:e46303 30361393 10.15252/embr.201846303 PMC6280791

[78] George CX, Samuel CE. 2011. Host response to polyomavirus infection is modulated by RNA adenosine deaminase ADAR1 but not by ADAR2. J. Virol 85:8338–47 21632755 10.1128/JVI.02666-10 PMC3147991

[79] Wang Y, Samuel CE. 2009. Adenosine deaminase ADAR1 increases gene expression at the translational level by decreasing protein kinase PKR-dependent eIF-2α phosphorylation. J. Mol. Biol 393:777–87 19733181 10.1016/j.jmb.2009.08.070 PMC2763985

[80] Bou-Nader C, Gordon JM, Henderson FE, Zhang J. 2019. The search for a PKR code-differential regulation of protein kinase R activity by diverse RNA and protein regulators. RNA 25:539–56 30770398 10.1261/rna.070169.118 PMC6467004

[81] Whitfield ZJ, Prasad AN, Ronk AJ, Kuzmin IV, Ilinykh PA, 2020. Species-specific evolution of Ebola virus during replication in human and bat cells. Cell Rep. 32:108028 32814037 10.1016/j.celrep.2020.108028 PMC7434439

[82] Zahn RC, Schelp I, Utermöhlen O, von Laer D. 2007. A-to-G hypermutation in the genome of lymphocytic choriomeningitis virus. J. Virol 81:457–64 17020943 10.1128/JVI.00067-06 PMC1797460

[83] Yanai M, Kojima S, Sakai M, Komorizono R, Tomonaga K, Makino A. 2020. ADAR2 is involved in self and nonself recognition of Borna disease virus genomic RNA in the nucleus. J. Virol 94:e01513–19 31852792 10.1128/JVI.01513-19 PMC7158724

[84] Cao Y, Cao R, Huang Y, Zhou H, Liu Y, 2018. A comprehensive study on cellular RNA editing activity in response to infections with different subtypes of influenza A viruses. BMC Genom. 19:925 10.1186/s12864-017-4330-1 PMC5780764 29363430

[85] Sarvestani ST, Tate MD, Moffat JM, Jacobi AM, Behlke MA, 2014. Inosine-mediated modulation of RNA sensing by Toll-like receptor 7 (TLR7) and TLR8. J. Virol 88:799–810 24227841 10.1128/JVI.01571-13 PMC3911656

[86] Suspène R, Petit V, Puyraimond-Zemmour D, Aynaud MM, Henry M, 2011. Double-stranded RNA adenosine deaminase ADAR-1-induced hypermutated genomes among inactivated seasonal influenza and live attenuated measles virus vaccines. J. Virol 85:2458–62 21159878 10.1128/JVI.02138-10 PMC3067779

[87] Vogel OA, Han J, Liang CY, Manicassamy S, Perez JT, Manicassamy B. 2020. The p150 isoform of ADAR1 blocks sustained RLR signaling and apoptosis during influenza virus infection. PLOS Pathog. 16:e1008842 32898178 10.1371/journal.ppat.1008842 PMC7500621

[88] Zhang T, Yin C, Boyd DF, Quarato G, Ingram JP, 2020. Influenza virus Z-RNAs induce ZBP1-mediated necroptosis. Cell 180:1115–29.e13 32200799 10.1016/j.cell.2020.02.050 PMC7153753

[89] Fensterl V, Chattopadhyay S, Sen GC. 2015. No love lost between viruses and interferons. Annu. Rev. Virol 2:549–72 26958928 10.1146/annurev-virology-100114-055249 PMC9549753

[90] de Chassey B, Aublin-Gex A, Ruggieri A, Meyniel-Schicklin L, Pradezynski F, 2013. The interactomes of influenza virus NS1 and NS2 proteins identify new host factors and provide insights for ADAR1 playing a supportive role in virus replication. PLOS Pathog. 9:e1003440 23853584 10.1371/journal.ppat.1003440 PMC3701712

[91] Biron CA, Nguyen KB, Pien GC. 2002. Innate immune responses to LCMV infections: natural killer cells and cytokines. Curr. Top. Microbiol. Immunol 263:7–27 11987821 10.1007/978-3-642-56055-2_2

[92] Suspène R, Renard M, Henry M, Guétard D, Puyraimond-Zemmour D, 2008. Inversing the natural hydrogen bonding rule to selectively amplify GC-rich ADAR-edited RNAs. Nucleic Acids Res. 36:e72 18515351 10.1093/nar/gkn295 PMC2475633

[93] Schoggins JW, Wilson SJ, Panis M, Murphy MY, Jones CT, 2011. A diverse range of gene products are effectors of the type I interferon antiviral response. Nature 472:481–85 21478870 10.1038/nature09907 PMC3409588

[94] Clavarino G, Cláudio N, Couderc T, Dalet A, Judith D, 2012. Induction of GADD34 is necessary for dsRNA-dependent interferon-β production and participates in the control of Chikungunya virus infection. PLOS Pathog. 8:e1002708 22615568 10.1371/journal.ppat.1002708 PMC3355096

[95] Khrustalev VV, Khrustaleva TA, Sharma N, Giri R. 2017. Mutational pressure in Zika virus: local ADAR-editing areas associated with pauses in translation and replication. Front. Cell. Infect. Microbiol 7:44 28275585 10.3389/fcimb.2017.00044 PMC5319961

[96] Piontkivska H, Frederick M, Miyamoto MM, Wayne ML. 2017. RNA editing by the host ADAR system affects the molecular evolution of the Zika virus. Ecol. Evol 7:4475–85 28649357 10.1002/ece3.3033 PMC5478085

[97] McGrath EL, Rossi SL, Gao J, Widen SG, Grant AC, 2017. Differential responses of human fetal brain neural stem cells to Zika virus infection. Stem Cell Rep. 8:715–27 10.1016/j.stemcr.2017.01.008 PMC5355569 28216147

[98] Zhou S, Yang C, Zhao F, Huang Y, Lin Y, 2019. Double-stranded RNA deaminase ADAR1 promotes the Zika virus replication by inhibiting the activation of protein kinase PKR. J. Biol. Chem 294:18168–80 31636123 10.1074/jbc.RA119.009113 PMC6885634

[99] Piontkivska H, Plonski NM, Miyamoto MM, Wayne ML. 2019. Explaining pathogenicity of congenital Zika and Guillain-Barré syndromes: Does dysregulation of RNA editing play a role? Bioessays 41:e1800239 31106880 10.1002/bies.201800239 PMC6699488

[100] Diosa-Toro M, Echavarría-Consuegra L, Flipse J, Fernández GJ, Kluiver J, 2017. MicroRNA profiling of human primary macrophages exposed to dengue virus identifies miRNA-3614-5p as antiviral and regulator of ADAR1 expression. PLOS Negl. Trop. Dis 11:e0005981 29045406 10.1371/journal.pntd.0005981 PMC5662241

[101] Paul D, Madan V, Bartenschlager R. 2014. Hepatitis C virus RNA replication and assembly: living on the fat of the land. Cell Host Microbe 16:569–79 25525790 10.1016/j.chom.2014.10.008 PMC7172941

[102] Luo S, Cassidy W, Jeffers L, Reddy KR, Bruno C, Howell CD. 2005. Interferon-stimulated gene expression in black and white hepatitis C patients during peginterferon alfa-2a combination therapy. Clin. Gastroenterol. Hepatol 3:499–506 15880320 10.1016/s1542-3565(04)00615-9

[103] Salvetat N, Van der Laan S, Vire B, Chimienti F, Cleophax S, 2019. RNA editing blood biomarkers for predicting mood alterations in HCV patients. J. Neurovirol 25:825–36 31332697 10.1007/s13365-019-00772-9 PMC6920238

[104] Garaigorta U, Chisari FV. 2009. Hepatitis C virus blocks interferon effector function by inducing protein kinase R phosphorylation. Cell Host Microbe 6:513–22 20006840 10.1016/j.chom.2009.11.004 PMC2905238

[105] Pujantell M, Franco S, Galván-Femenía I, Badia R, Castellví M, 2018. ADAR1 affects HCV infection by modulating innate immune response. Antivir. Res 156:116–27 29906476 10.1016/j.antiviral.2018.05.012

[106] Taylor DR, Puig M, Darnell ME, Mihalik K, Feinstone SM. 2005. New antiviral pathway that mediates hepatitis C virus replicon interferon sensitivity through ADAR1. J. Virol 79:6291–98 15858013 10.1128/JVI.79.10.6291-6298.2005 PMC1091666

[107] Li Y, Masaki T, Shimakami T, Lemon SM. 2014. hnRNP L and NF90 interact with hepatitis C virus 5’-terminal untranslated RNA and promote efficient replication. J. Virol 88:7199–209 24719423 10.1128/JVI.00225-14 PMC4054450

[108] Liu CX, Li X, Nan F, Jiang S, Gao X, 2019. Structure and degradation of circular RNAs regulate PKR activation in innate immunity. Cell 177:865–80.e21 31031002 10.1016/j.cell.2019.03.046

[109] Ivanov A, Memczak S, Wyler E, Torti F, Porath HT, 2015. Analysis of intron sequences reveals hallmarks of circular RNA biogenesis in animals. Cell Rep. 10:170–77 25558066 10.1016/j.celrep.2014.12.019

[110] Sa Ribero M, Jouvenet N, Dreux M, Nisole S. 2020. Interplay between SARS-CoV-2 and the type I interferon response. PLOS Pathog. 16:e1008737 32726355 10.1371/journal.ppat.1008737 PMC7390284

[111] Niemeyer D, Zillinger T, Muth D, Zielecki F, Horvath G, 2013. Middle East respiratory syndrome coronavirus accessory protein 4a is a type I interferon antagonist. J. Virol 87:12489–95 24027320 10.1128/JVI.01845-13 PMC3807936

[112] Thornbrough JM, Jha BK, Yount B, Goldstein SA, Li Y, 2016. Middle East respiratory syndrome coronavirus NS4b protein inhibits host RNase L activation. mBio 7:e00258 27025250 10.1128/mBio.00258-16 PMC4817253

[113] Zhao L, Jha BK, Wu A, Elliott R, Ziebuhr J, 2012. Antagonism of the interferon-induced OAS-RNase L pathway by murine coronavirus ns2 protein is required for virus replication and liver pathology. Cell Host Microbe 11:607–16 22704621 10.1016/j.chom.2012.04.011 PMC3377938

[114] Deng X, Hackbart M, Mettelman RC, O’Brien A, Mielech AM, 2017. Coronavirus nonstructural protein 15 mediates evasion of dsRNA sensors and limits apoptosis in macrophages. PNAS 114:E4251–60 28484023 10.1073/pnas.1618310114 PMC5448190

[115] Di Giorgio S, Martignano F, Torcia MG, Mattiuz G, Conticello SG. 2020. Evidence for host-dependent RNA editing in the transcriptome of SARS-CoV-2. Sci. Adv 6:eabb5813 32596474 10.1126/sciadv.abb5813 PMC7299625

[116] Wang R, Hozumi Y, Zheng YH, Yin C, Wei GW. 2020. Host immune response driving SARS-CoV-2 evolution. Viruses 12:1095 32992592 10.3390/v12101095 PMC7599751

[117] Tao Y, Farsetta DL, Nibert ML, Harrison SC. 2002. RNA synthesis in a cage—structural studies of reovirus polymerase λ3. Cell 111:733–45 12464184 10.1016/s0092-8674(02)01110-8

[118] Liu Y, Wolff KC, Jacobs BL, Samuel CE. 2001. Vaccinia virus E3L interferon resistance protein inhibits the interferon-induced adenosine deaminase A-to-I editing activity. Virology 289:378–87 11689059 10.1006/viro.2001.1154

[119] Kumar M, Carmichael GG. 1997. Nuclear antisense RNA induces extensive adenosine modifications and nuclear retention of target transcripts. PNAS 94:3542–47 9108012 10.1073/pnas.94.8.3542 PMC20475

[120] Gu R, Zhang Z, DeCerbo JN, Carmichael GG. 2009. Gene regulation by sense-antisense overlap of polyadenylation signals. RNA 15:1154–63 19390116 10.1261/rna.1608909 PMC2685520

[121] Chiang C, Pauli EK, Biryukov J, Feister KF, Meng M, 2018. The human papillomavirus E6 oncoprotein targets USP15 and TRIM25 to suppress RIG-I-mediated innate immune signaling. J. Virol 92:e01737–17 29263274 10.1128/JVI.01737-17 PMC5827370

[122] Pujantell M, Badia R, Galván-Femenía I, Garcia-Vidal E, de Cid R, 2019. ADAR1 function affects HPV replication and is associated to recurrent human papillomavirus-induced dysplasia in HIV coinfected individuals. Sci. Rep 9:19848 31882741 10.1038/s41598-019-56422-x PMC6934649

[123] Diao MK, Liu CY, Liu HW, Li JT, Li F, 2015. Integrated HPV genomes tend to integrate in gene desert areas in the CaSki, HeLa, and SiHa cervical cancer cell lines. Life Sci. 127:46–52 25747255 10.1016/j.lfs.2015.01.039

[124] Gandy SZ, Linnstaedt SD, Muralidhar S, Cashman KA, Rosenthal LJ, Casey JL. 2007. RNA editing of the human herpesvirus 8 kaposin transcript eliminates its transforming activity and is induced during lytic replication. J. Virol 81:13544–51 17913828 10.1128/JVI.01521-07 PMC2168827

[125] Iizasa H, Wulff BE, Alla NR, Maragkakis M, Megraw M, 2010. Editing of Epstein-Barr virus-encoded BART6 microRNAs controls their dicer targeting and consequently affects viral latency. J. Biol. Chem 285:33358–70 20716523 10.1074/jbc.M110.138362 PMC2963350

[126] Cao S, Moss W, O’Grady T, Concha M, Strong MJ, 2015. New noncoding lytic transcripts derived from the Epstein-Barr virus latency origin of replication, oriP, are hyperedited, bind the paraspeckle protein, NONO/p54nrb, and support viral lytic transcription. J. Virol 89:7120–32 25926645 10.1128/JVI.00608-15 PMC4473578

[127] Rosani U, Bai CM, Maso L, Shapiro M, Abbadi M, 2019. A-to-I editing of *Malacoherpesviridae* RNAs supports the antiviral role of ADAR1 in mollusks. BMC Evol. Biol 19:149 31337330 10.1186/s12862-019-1472-6 PMC6651903

[128] Lei T, Yuen KS, Tsao SW, Chen H, Kok KH, Jin DY. 2013. Perturbation of biogenesis and targeting of Epstein-Barr virus-encoded miR-BART3 microRNA by adenosine-to-inosine editing. J. Gen. Virol 94:2739–44 24045110 10.1099/vir.0.056226-0

[129] Ishiguro S, Galipon J, Ishii R, Suzuki Y, Kondo S, 2018. Base-pairing probability in the microRNA stem region affects the binding and editing specificity of human A-to-I editing enzymes ADAR1-p110 and ADAR2. RNA Biol. 15:976–89 29950133 10.1080/15476286.2018.1486658 PMC6161747

[130] Pinto Y, Buchumenski I, Levanon EY, Eisenberg E. 2018. Human cancer tissues exhibit reduced A-to-I editing of miRNAs coupled with elevated editing of their targets. Nucleic Acids Res. 46:71–82 29165639 10.1093/nar/gkx1176 PMC5758889

[131] Kawahara Y, Megraw M, Kreider E, Iizasa H, Valente L, 2008. Frequency and fate of microRNA editing in human brain. Nucleic Acids Res. 36:5270–80 18684997 10.1093/nar/gkn479 PMC2532740

[132] Paul D, Sinha AN, Ray A, Lal M, Nayak S, 2017. A-to-I editing in human miRNAs is enriched in seed sequence, influenced by sequence contexts and significantly hypoedited in glioblastoma multiforme. Sci. Rep 7:2466 28550310 10.1038/s41598-017-02397-6 PMC5446428

[133] Damania B. 2004. Oncogenic γ-herpesviruses: comparison of viral proteins involved in tumorigenesis. Nat. Rev. Microbiol 2:656–68 15263900 10.1038/nrmicro958

[134] Pfeffer S, Zavolan M, Grässer FA, Chien M, Russo JJ, 2004. Identification of virus-encoded microRNAs. Science 304:734–36 15118162 10.1126/science.1096781

[135] Arias C, Weisburd B, Stern-Ginossar N, Mercier A, Madrid AS, 2014. KSHV 2.0: A comprehensive annotation of the Kaposi’s sarcoma-associated herpesvirus genome using next-generation sequencing reveals novel genomic and functional features. PLOS Pathog. 10:e1003847 24453964 10.1371/journal.ppat.1003847 PMC3894221

[136] Sommer B, Köhler M, Sprengel R, Seeburg PH. 1991. RNA editing in brain controls a determinant of ion flow in glutamate-gated channels. Cell 67:11–19 1717158 10.1016/0092-8674(91)90568-j

[137] Burns CM, Chu H, Rueter SM, Hutchinson LK, Canton H, 1997. Regulation of serotonin-2C receptor G-protein coupling by RNA editing. Nature 387:303–8 9153397 10.1038/387303a0

[138] Liu Y, Samuel CE. 1999. Editing of glutamate receptor subunit B pre-mRNA by splice-site variants of interferon-inducible double-stranded RNA-specific adenosine deaminase ADAR1. J. Biol. Chem 274:5070–77 9988754 10.1074/jbc.274.8.5070

[139] Liu Y, Emeson RB, Samuel CE. 1999. Serotonin-2C receptor pre-mRNA editing in rat brain and in vitro by splice site variants of the interferon-inducible double-stranded RNA-specific adenosine deaminase ADAR1. J. Biol. Chem 274:18351–58 10373439 10.1074/jbc.274.26.18351

[140] Lei M, Liu Y, Samuel CE. 1998. Adenovirus VAI RNA antagonizes the RNA-editing activity of the ADAR adenosine deaminase. Virology 245:188–96 9636358 10.1006/viro.1998.9162

[141] Kitajewski J, Schneider RJ, Safer B, Munemitsu SM, Samuel CE, 1986. Adenovirus VAI RNA antagonizes the antiviral action of interferon by preventing activation of the interferon-induced eIF-2α kinase. Cell 45:195–200 3698097 10.1016/0092-8674(86)90383-1

[142] Langland JO, Jacobs BL. 2004. Inhibition of PKR by vaccinia virus: role of the N- and C-terminal domains of E3L. Virology 324:419–29 15207627 10.1016/j.virol.2004.03.012

[143] Zhang P, Jacobs BL, Samuel CE. 2008. Loss of protein kinase PKR expression in human HeLa cells complements the vaccinia virus E3L deletion mutant phenotype by restoration of viral protein synthesis. J. Virol 82:840–48 17959656 10.1128/JVI.01891-07 PMC2224564

[144] Kim YG, Muralinath M, Brandt T, Pearcy M, Hauns K, 2003. A role for Z-DNA binding in vaccinia virus pathogenesis. PNAS 100:6974–79 12777633 10.1073/pnas.0431131100 PMC165815

[145] Liao GR, Tseng YY, Tseng CY, Lin FY, Yamada Y, 2019. Adenosine deaminase acting on RNA 1 associates with Orf virus OV20.0 and enhances viral replication. J. Virol 93:e01912–18 30651363 10.1128/JVI.01912-18 PMC6430553

[146] Bockmann JH, Stadler D, Xia Y, Ko C, Wettengel JM, 2019. Comparative analysis of the antiviral effects mediated by type I and III interferons in hepatitis B virus-infected hepatocytes. J. Infect. Dis 220:567–77 30923817 10.1093/infdis/jiz143

[147] Liu G, Ma X, Wang Z, Wakae K, Yuan Y, 2019. Adenosine deaminase acting on RNA-1 (ADAR1) inhibits hepatitis B virus (HBV) replication by enhancing microRNA-122 processing. J. Biol. Chem 294:14043–54 31366735 10.1074/jbc.RA119.007970 PMC6755794

[148] Yuan L, Jia Q, Yang S, Idris NFB, Li Y, 2020. ADAR1 promotes HBV replication through its deaminase domain. Front. Biosci. (Landmark Ed.) 25:710–21 31585913 10.2741/4830

[149] Wu X, Xin Z, Zhu X, Pan L, Li Z, 2012. Polymorphisms in ADAR1 gene affect response to interferon alpha based therapy for chronic hepatitis B in Han Chinese. Antivir. Res 94:272–75 22449829 10.1016/j.antiviral.2012.03.004

[150] Wu X, Shi W, Wu J, Zhu X, Chen K, 2014. A functional polymorphism in ADAR1 gene affects HBsAg seroclearance both spontaneously and interferon induced. Liver Int. 34:1560–65 24351124 10.1111/liv.12444

[151] Jung S, Altstetter SM, Protzer U. 2020. Innate immune recognition and modulation in hepatitis D virus infection. World J. Gastroenterol 26:2781–91 32550754 10.3748/wjg.v26.i21.2781 PMC7284172

[152] Jayan GC, Casey JL. 2002. Inhibition of hepatitis delta virus RNA editing by short inhibitory RNA-mediated knockdown of ADAR1 but not ADAR2 expression. J. Virol 76:12399–404 12414985 10.1128/JVI.76.23.12399-12404.2002 PMC136899

[153] Wong SK, Lazinski DW. 2002. Replicating hepatitis delta virus RNA is edited in the nucleus by the small form of ADAR1. PNAS 99:15118–23 12399548 10.1073/pnas.232416799 PMC137553

[154] Casey JL. 2006. RNA editing in hepatitis delta virus. Curr. Top. Microbiol. Immunol 307:67–89 16903221 10.1007/3-540-29802-9_4

[155] Jayan GC, Casey JL. 2002. Increased RNA editing and inhibition of hepatitis delta virus replication by high-level expression of ADAR1 and ADAR2. J. Virol 76:3819–27 11907222 10.1128/JVI.76.8.3819-3827.2002 PMC136091

[156] Radetskyy R, Daher A, Gatignol A. 2018. ADAR1 and PKR, interferon stimulated genes with clashing effects on HIV-1 replication. Cytokine Growth Factor Rev. 40:48–58 29625900 10.1016/j.cytogfr.2018.03.007

[157] Phuphuakrat A, Kraiwong R, Boonarkart C, Lauhakirti D, Lee TH, Auewarakul P. 2008. Double-stranded RNA adenosine deaminases enhance expression of human immunodeficiency virus type 1 proteins. J. Virol 82:10864–72 18753201 10.1128/JVI.00238-08 PMC2573204

[158] Clerzius G, Gélinas JF, Daher A, Bonnet M, Meurs EF, Gatignol A. 2009. ADAR1 interacts with PKR during human immunodeficiency virus infection of lymphocytes and contributes to viral replication. J. Virol 83:10119–28 19605474 10.1128/JVI.02457-08 PMC2747994

[159] Doria M, Neri F, Gallo A, Farace MG, Michienzi A. 2009. Editing of HIV-1 RNA by the double-stranded RNA deaminase ADAR1 stimulates viral infection. Nucleic Acids Res. 37:5848–58 19651874 10.1093/nar/gkp604 PMC2761272

[160] Pujantell M, Riveira-Muñoz E, Badia R, Castellví M, Garcia-Vidal E, 2017. RNA editing by ADAR1 regulates innate and antiviral immune functions in primary macrophages. Sci. Rep 7:13339 29042669 10.1038/s41598-017-13580-0 PMC5645456

[161] Doria M, Tomaselli S, Neri F, Ciafrè SA, Farace MG, 2011. ADAR2 editing enzyme is a novel human immunodeficiency virus-1 proviral factor. J. Gen. Virol 92:1228–32 21289159 10.1099/vir.0.028043-0

[162] Gélinas JF, Clerzius G, Shaw E, Gatignol A. 2011. Enhancement of replication of RNA viruses by ADAR1 via RNA editing and inhibition of RNA-activated protein kinase. J. Virol 85:8460–66 21490091 10.1128/JVI.00240-11 PMC3165853

[163] Chukwurah E, Handy I, Patel RC. 2017. ADAR1 and PACT contribute to efficient translation of transcripts containing HIV-1 trans-activating response (TAR) element. Biochem. J 474:1241–57 28167698 10.1042/BCJ20160964 PMC5363390

[164] Orecchini E, Federico M, Doria M, Arenaccio C, Giuliani E, 2015. The ADAR1 editing enzyme is encapsidated into HIV-1 virions. Virology 485:475–80 26363218 10.1016/j.virol.2015.07.027

[165] Harris RS, Hultquist JF, Evans DT. 2012. The restriction factors of human immunodeficiency virus. J. Biol. Chem 287:40875–83 23043100 10.1074/jbc.R112.416925 PMC3510791

[166] Zheng Y, Lorenzo C, Beal PA. 2017. DNA editing in DNA/RNA hybrids by adenosine deaminases that act on RNA. Nucleic Acids Res. 45:3369–77 28132026 10.1093/nar/gkx050 PMC5389660

[167] Orecchini E, Frassinelli L, Galardi S, Ciafrè SA, Michienzi A. 2018. Post-transcriptional regulation of LINE-1 retrotransposition by AID/APOBEC and ADAR deaminases. Chromosome Res. 26:45–59 29396793 10.1007/s10577-018-9572-5

[168] Orecchini E, Doria M, Antonioni A, Galardi S, Ciafrè SA, 2017. ADAR1 restricts LINE-1 retrotransposition. Nucleic Acids Res. 45:155–68 27658966 10.1093/nar/gkw834 PMC5224506

[169] Hajjar AM, Linial ML. 1995. Modification of retroviral RNA by double-stranded RNA adenosine deaminase. J. Virol 69:5878–82 7543593 10.1128/jvi.69.9.5878-5882.1995 PMC189466

[170] Felder MP, Laugier D, Yatsula B, Dezélée P, Calothy G, Marx M. 1994. Functional and biological properties of an avian variant long terminal repeat containing multiple A to G conversions in the U3 sequence. J. Virol 68:4759–67 8035477 10.1128/jvi.68.8.4759-4767.1994 PMC236415

[171] Ko NL, Birlouez E, Wain-Hobson S, Mahieux R, Vartanian JP. 2012. Hyperediting of human T-cell leukemia virus type 2 and simian T-cell leukemia virus type 3 by the dsRNA adenosine deaminase ADAR-1. J. Gen. Virol 93:2646–51 22993189 10.1099/vir.0.045146-0 PMC4091295

[172] Cachat A, Alais S, Chevalier SA, Journo C, Fusil F, 2014. ADAR1 enhances HTLV-1 and HTLV-2 replication through inhibition of PKR activity. Retrovirology 11:93 25389016 10.1186/s12977-014-0093-9 PMC4245799

[173] Li S, Min JY, Krug RM, Sen GC. 2006. Binding of the influenza A virus NS1 protein to PKR mediates the inhibition of its activation by either PACT or double-stranded RNA. Virology 349:13–21 16466763 10.1016/j.virol.2006.01.005

[174] Chilibeck KA, Wu T, Liang C, Schellenberg MJ, Gesner EM, 2006. FRET analysis of *in vivo* dimerization by RNA-editing enzymes. J. Biol. Chem 281:16530–35 16618704 10.1074/jbc.M511831200

[175] Thuy-Boun AS, Thomas JM, Grajo HL, Palumbo CM, Park S, 2020. Asymmetric dimerization of adenosine deaminase acting on RNA facilitates substrate recognition. Nucleic Acids Res. 48:7958–72 32597966 10.1093/nar/gkaa532 PMC7641318

[176] Tariq A, Garncarz W, Handl C, Balik A, Pusch O, Jantsch MF. 2013. RNA-interacting proteins act as site-specific repressors of ADAR2-mediated RNA editing and fluctuate upon neuronal stimulation. Nucleic Acids Res. 41:2581–93 23275536 10.1093/nar/gks1353 PMC3575830

[177] Shanmugam R, Zhang F, Srinivasan H, Richard JLC, Liu KI, 2018. SRSF9 selectively represses ADAR2-mediated editing of brain-specific sites in primates. Nucleic Acids Res. 46:7379–95 29992293 10.1093/nar/gky615 PMC6101530

[178] Quinones-Valdez G, Tran SS, Jun HI, Bahn JH, Yang EW, 2019. Regulation of RNA editing by RNA-binding proteins in human cells. Commun. Biol 2:19 30652130 10.1038/s42003-018-0271-8 PMC6331435

[179] Aktaş T, Avşar Ilık İ, Maticzka D, Bhardwaj V, Pessoa Rodrigues C, 2017. DHX9 suppresses RNA processing defects originating from the *Alu* invasion of the human genome. Nature 544:115–19 28355180 10.1038/nature21715

[180] Freund EC, Sapiro AL, Li Q, Linder S, Moresco JJ, 2020. Unbiased identification of *trans* regulators of ADAR and A-to-I RNA editing. Cell Rep. 31:107656 32433965 10.1016/j.celrep.2020.107656 PMC7306178

[181] Sapiro AL, Freund EC, Restrepo L, Qiao HH, Bhate A, 2020. Zinc finger RNA-binding protein Zn72D regulates ADAR-mediated RNA editing in neurons. Cell Rep. 31:107654 32433963 10.1016/j.celrep.2020.107654 PMC7306179

[182] van den Hoogen BG, van Boheemen S, de Rijck J, van Nieuwkoop S, Smith DJ, 2014. Excessive production and extreme editing of human metapneumovirus defective interfering RNA is associated with type I IFN induction. J. Gen. Virol 95:1625–33 24760760 10.1099/vir.0.066100-0 PMC4103063

[183] Nguyen TA, Smith BRC, Tate MD, Belz GT, Barrios MH, 2017. SIDT2 transports extracellular dsRNA into the cytoplasm for innate immune recognition. Immunity 47:498–509.e6 28916264 10.1016/j.immuni.2017.08.007 PMC5679266

[184] Nguyen TA, Smith BRC, Elgass KD, Creed SJ, Cheung S, 2019. SIDT1 localizes to endolysosomes and mediates double-stranded RNA transport into the cytoplasm. J. Immunol 202:3483–92 31061008 10.4049/jimmunol.1801369

